# Mechanistic Insights from the Crystal Structure and Computational Analysis of the Radical SAM Deaminase DesII

**DOI:** 10.1002/advs.202403494

**Published:** 2024-06-28

**Authors:** Xueli Hou, Jianqiang Feng, Joseph Livy Franklin, Ryan Russo, Zhiyong Guo, Jiahai Zhou, Jin‐Ming Gao, Hung‐wen Liu, Binju Wang

**Affiliations:** ^1^ Shaanxi Key Laboratory of Natural Products & Chemical Biology College of Chemistry & Pharmacy Northwest A&F University Yangling Shaanxi 712100 China; ^2^ Key Laboratory of Quantitative Synthetic Biology Shenzhen Institute of Synthetic Biology Shenzhen Institutes of Advanced Technology Chinese Academy of Sciences Shenzhen 518055 China; ^3^ State Key Laboratory of Physical Chemistry of Solid Surfaces and Fujian Provincial Key Laboratory of Theoretical and Computational Chemistry College of Chemistry and Chemical Engineering and Innovation Laboratory for Sciences and Technologies of Energy Materials of Fujian Province (IKKEM) Xiamen University Xiamen 361005 China; ^4^ Division of Chemical Biology & Medicinal Chemistry College of Pharmacy University of Texas at Austin Austin TX 78712 USA; ^5^ State Key Laboratory of Biocatalysis and Enzyme Engineering Hubei Key Laboratory of Industrial Biotechnology School of Life Sciences Hubei University Wuhan 430062 China; ^6^ School of Food Science and Pharmaceutical Engineering Nanjing Normal University Nanjing 210023 China; ^7^ Department of Chemistry University of Texas at Austin Austin TX 78712 USA

**Keywords:** catalytic mechanism, computational analysis, crystal structure, radical SAM enzyme

## Abstract

Radical *S*‐adenosyl‐L‐methionine (SAM) enzymes couple the reductive cleavage of SAM to radical‐mediated transformations that have proven to be quite broad in scope. DesII is one such enzyme from the biosynthetic pathway of TDP‐desosamine where it catalyzes a radical‐mediated deamination. Previous studies have suggested that this reaction proceeds via direct elimination of ammonia from an α‐hydroxyalkyl radical or its conjugate base (i.e., a ketyl radical) rather than 1,2‐migration of the amino group to form a carbinolamine radical intermediate. However, without a crystal structure, the active site features responsible for this chemistry have remained largely unknown. The crystallographic studies described herein help to fill this gap by providing a structural description of the DesII active site. Computational analyses based on the solved crystal structure are consistent with direct elimination and indicate that an active site glutamate residue likely serves as a general base to promote deprotonation of the α‐hydroxyalkyl radical intermediate and elimination of the ammonia group.

## Graphical abstract

DesII is a radical *S*‐adenosyl‐l‐methione enzyme that catalyzes the radical‐mediated deamination of TDP‐4‐amino‐4,6‐dideoxy‐d‐glucose to TDP‐3‐keto‐4,6‐dideoxy‐d‐glucose. Computational analyses based on the binary DesII·SAM crystal structure indicate that an active site glutamate residue serves as a general base to promote the deprotoantion of the α‐hydroxyalkyl radical intermediate and the elimination of the ammonia group.

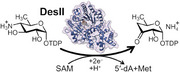

## 1 Introduction

**Figure 1:**
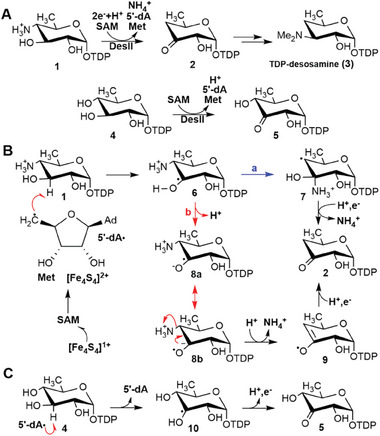
A) DesII is a radical SAM deaminase that catalyzes the deamination of **1** to **2** during the biosynthesis of **3** as well as the dehydrogenation of **4**. B) DesII catalyzed deamination can be broadly classified into one of two mechanisms depending on the intermediacy of a carbinolamine radical (**7**). C) DesII catalyzed dehydrogenation of **4** also involves the C3′ *α*‐hydroxyalkyl radical **10**. Abbreviations: SAM, *S*‐adenosyl‐l‐methionine; 5′‐dA, 5′‐deoxyadenosine; 5′‐dA•, 5′‐deoxyadenosyl radical; Met, l‐methionine; TDP, thymidine diphosphate.

DesII is a radical *S*‐adenosyl‐l‐methione (SAM) enzyme that catalyzes the radical‐mediated deamination of TDP‐4‐amino‐4,6‐dideoxy‐d‐glucose (**1**) to TDP‐3‐keto‐4,6‐dideoxy‐d‐glucose (**2**) during the biosynthesis of TDP‐desosamine (**3**, **Figure** **1**A).^[^ ^1^, ^2^, ^3^, ^4^ ^]^ Like other radical SAM enzymes,^[^ ^5^, ^6^ ^]^ the catalytic cycle of DesII is characterized by initial single electron transfer from a reduced, active site [Fe_4_S_4_]^+^ cluster to SAM (Figure 1B). This leads to the reductive cleavage of SAM yielding a 5′‐deoxyadenosyl radical (5′‐dA•) or its equivalent that abstracts a hydrogen atom from the C3′ position of **1**. The resulting *α*‐hydroxyalkyl radical (**6**) can then undergo the radical‐mediated elimination of ammonia and subsequent reduction to the carbonyl product (**2**). This chemistry is similar to that of the adenosyl‐cobalamin (AdoCbl) dependent ethanolamine ammonia lyase (EAL);^[^ ^7^ ^]^ however, in neither case has a definitive mechanism of catalysis been established.

Mechanistic hypotheses for DesII and EAL can broadly be classified according to whether they involve a carbinolamine radical's intermediacy (e.g., **7**). In other words, the *α*‐hydroxyalkyl radical (**6**) may first undergo a 1,2‐migration of the *β*‐amino group to yield an intermediate such as **7** (Figure 1B, pathway **a**), producing only **2** after reduction and elimination. A radical‐mediated migration mechanism has generally been favored for EAL^[^ ^7^, ^8^, ^9^, ^10^, ^11^ ^]^ and is also consistent with current models of AdoCbl‐dependent diol‐dehydratase catalysis.^[^ ^12^, ^13^ ^]^ However, experimental work on DesII,^[^ ^14^, ^15^ ^]^ radical SAM diol‐dehydratases,^[^ ^16^, ^17^, ^18^ ^]^ the ribonucleotide reductases,^[^ ^19^, ^20^ ^]^ the glycyl radical enzyme propandiol dehydratase^[^ ^21^ ^]^ and several other radical‐mediated lyases^[^ ^22^, ^23^ ^]^ has instead pointed to an alternative mechanism that does not involve a 1,2‐migration. In this case, the *β*‐nucleofuge is directly eliminated from either the *α*‐hydroxyalkyl radical or its conjugate base (i.e., a ketyl radical, **8**) to yield an enol radical (**9**) that subsequently undergoes reduction to the product (Figure 1B, pathway **b**). Consequently, enzymes catalyzing radical‐mediated deamination or dehydration reactions may not be restricted to a single mechanistic paradigm.

A peculiar feature of DesII is that it can also accept TDP‐d‐quinovose (**4**) as an alternative substrate in which a hydroxyl group has been replaced by the C4′‐amine.^[^ ^2^ ^]^ Despite the apparent similarities between radical‐mediated dehydratases and deaminases, DesII does not catalyze dehydration of (**4**) but rather its dehydrogenation (**4** → **5**, Figure 1A). This has led to speculation about what mediates partitioning of the C3′ substrate radical (i.e., **6** or **10**) between the lyase and dehydrogenase pathways once generated in the DesII active site.^[^ ^4^ ^]^ EPR characterization of the freeze‐trapped substrate radical derived from **4** (i.e., **10**, Figure 1C) implied a roughly clinal relationship between the partially‐filled *p*‐orbital at C3′ and the C4′─OH bond.^[^ ^24^ ^]^ Consequently, it has been proposed that a specific geometry may be required to optimize overlap between the partially filled *p*‐orbital and adjacent C─OH bond to facilitate elimination either directly or via 1,2‐migration instead of dehydrogenation.^[^ ^4^ ^]^ This mechanism follows the classic geometric rules governing the catalytic outcome of triosephosphate isomerase^[^ ^25^ ^]^ generalized to highly reactive radical intermediates.

The importance of radical geometry on catalytic partitioning has garnered both experimental and computational support. For example, changing the C4′ stereochemistry of TDP‐d‐quinovose (**4**) to yield TDP‐d‐fucose results in a DesII substrate that undergoes at least partial dehydration.^[^ ^26^ ^]^ Likewise, a careful study of the radical SAM dehydratase AprD4 has demonstrated an interplay between acid‐base catalysis and substrate conformational changes to minimize the activation energy for dehydration.^[^ ^16^ ^]^ Moreover, the importance of radical geometry as a factor in determining product distributions has also been noted in nonenzymatic carbohydrate reactions mediated by radicals.^[^ ^27^ ^]^ Nevertheless, other factors such as leaving group activation and proton transfer are likely also at work if not dominant in deciding the fate of vital radical intermediates in an enzyme active site.

The present work reports the 2.33 Å crystal structure of DesII as a binary complex with SAM bound in the active site. This structure is a foundation for QM/MM and molecular dynamics computations to compare the energetics of proposed mechanisms for DesII catalysis. Consistent with previous experimental results, the minimum energy pathway for deamination of **1** involves direct elimination as opposed to radical‐mediated 1,2‐migration of the C4′‐amino group. Moreover, this elimination appears to be facilitated by an active site glutamate residue that is proposed to engage in H‐bonding interactions with the C3′ hydroxyl and C4' ammonium moieties of **1** to facilitate net proton transfer from the *α*‐hydroxyalkyl radical to the eliminated ammonia. A similar analysis on the dehydrogenation substrate **4** indicates that proton‐coupled electron transfer to the [Fe_4_S_4_]^2+^ cluster is energetically more feasible than dehydration via direct elimination or migration. The critical factor deciding the reaction outcome appears to be predominantly electrostatic such that the positively charged C4′‐ammonium moiety disfavors proton‐coupled electron transfer from the substrate radical.

## 2 Results and Discussion

### 2.1 Crystal Structure of DesII

**Figure 2:**
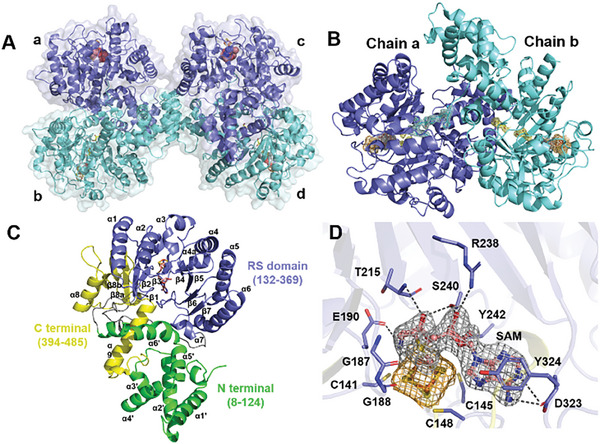
A) Asymmetric unit of the DesII·SAM binary complex. B) Local structure of the binary complex with chain a in blue and chain b in cyan demonstrating occupancy of the chain a active site by the *C*‐terminal His_6_‐tag of chain b. C) Secondary and tertiary structure of each DesII monomer illustrating the three domains. D) SAM (pink) is bound in the DesII active site via contacts with the unique iron in the [Fe_4_S_4_] cluster as well as the GGE (G187, G188, L189 & E190), ribose (R238, S240, & Y242), GXIXGXXE and *β*_6_ motifs (D323 & Y324). Simulated annealing omit maps are contoured at 2.0 *σ* ([Fe4S4] cluster in orange mesh, SAM in gray mesh, Met in lightgray mesh, glycerol in yellow mesh, and His6‐tag of chain b in cyan mesh).

The crystal structure of DesII from *Streptomyces venezuelae* was determined by single anomalous diffraction using a selenomethionine derivative. The crystal diffracted to 2.33 Å resolution (Table S1, Supporting Information, PDB 8HZV) and demonstrated an overall quaternary structure consisting of four monomeric units (chains a–d, **Figure** **2**A). The *N*‐termini were found to be disordered such that seven residues from chains a and b and nine residues from chains c and d were not included in the final model. Amino acid residues Y324–Q335 were missing in chains b and d. A single molecule of SAM was found coordinated to the [Fe_4_S_4_] cluster in chains a and c with methionine bound in the active sites of chains b and d. The *C*‐terminal His_6_‐tags of chains b and d were found to occupy the active site pockets of chains a and c, respectively (Figure 2B). Two glycerol molecules were also found in the active sites of chains b and d, likely due to cryoprotection with a 5% glycerol solution before flash‐freezing in liquid nitrogen (Section S1.4, Supporting Information).

Each monomeric unit comprises three domains: an *N*‐terminal domain of six *α*‐helices, a central radical SAM (RS) domain, and a *C*‐terminal domain consisting of a *β*‐hairpin and two *α*‐helices (Figure 2C). The RS domain (residues S132–L369) coordinates the single [Fe_4_S_4_] cluster via residues C141, C145, and C148, which make up the CX_3_CX_2_C motif.^[^ ^28^ ^]^ Four other previously identified SAM binding motifs including the GGE, ribose, GXIXGXXE, and *β*_6_ motif^[^ ^6^, ^16^, ^29^ ^]^ are also conserved in DesII. As displayed in Figure 2D, the GGE motif (G187–E190) interacts with the Met moiety of SAM via H‐bonding interactions. In contrast, the R238, S240, and Y242 residues at the end of the *β*_4_ strand H‐bond with the SAM ribose moiety to form the “ribose motif” originally described in BioB.^[^ ^29^, ^30^ ^]^ The GXIXGXXE motif provides hydrophobic contacts to the adenine ring of SAM,^[^ ^29^, ^31^ ^]^ which is further stabilized by backbone interactions with a polar residue in *α*_5_ and hydrophobic interactions with Y324 as found in other radical SAM enzymes.^[^ ^18^ ^]^ Residue D323 in the loop behind the *β*_6_ strand provides two backbone interactions with N1 and N6 of the adenine base, which is another feature of the *β*_6_ motif.^[^ ^29^ ^]^

**Figure 3:**
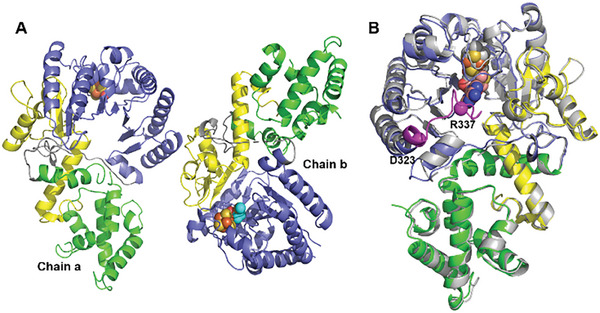
A) Asymmetric unit of DesII without SAM bound contains two molecular units labeled a and b. B) Superimposition (rmsd 0.533 Å) of unbound DesII (grey) with the DesII·SAM complex (colored as in Figure 2C). The flexible loop between D323 and R337 is marked in magenta (*α*‐carbons are highlighted as magenta spheres).

Addition of TDP‐4‐amino‐4,6‐dideoxy‐d‐glucose (**1**) or TDP‐d‐quinovose (**4**) during crystallization screening did not result in the acquisition of a ternary complex structure in either case, and an effort was made to remove the *C*‐terminal His_6_‐tag found to occupy the binding pockets of chains a and c. Therefore, a second DesII expression construct was generated by inserting the *desII* gene into the pSJ5 expression vector containing a TrxA fusion partner, His_8_‐tag, and TEV site. This produced soluble, high‐purity protein without the *N*‐terminal His‐tag after purification by Ni‐NTA extraction and TEV enzyme digestion. The corresponding crystals were grown in 0.1 m MES monohydrate containing 20% PEG 6000 and 1.0 m LiCl at pH 6.0. The crystal structure (PDB ID: 8HZY) was solved to a resolution of 2.04 Å by molecular replacement using chain a of the binary complex as the search model. The asymmetric unit consisted of two DesII monomers, neither bound SAM; instead, the fourth iron of the [Fe_4_S_4_] cluster in one monomer coordinated with a water molecule and the other with methionine (**Figure** **3**A). Comparison with the binary DesII·SAM structure (rmsd 0.533 Å) revealed that the flexible loop between residues D323 and E337, which interact with the adenine moiety of SAM in the binary structure, was also missing from the density map (Figure 3B). Addition of **1** or **4** to the crystallization media did not result in substrate‐bound complexes.

### 2.2 Mechanism of Deamination

**Figure 4:**
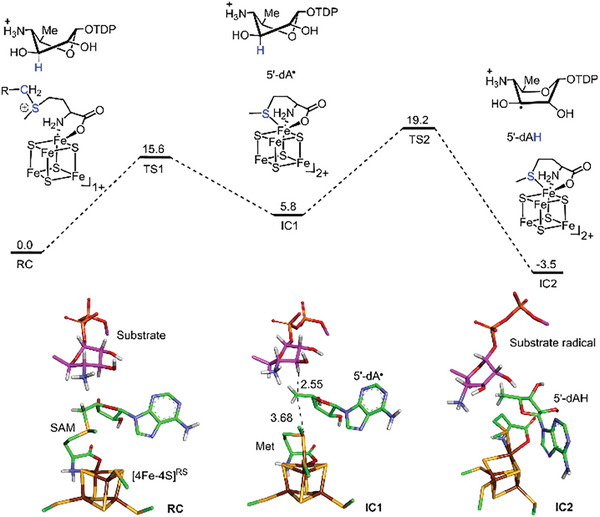
QM(TPSSh/def2‐TZVP)/MM calculated energy profile (kcal mol^−1^) for the reductive S−C cleavage of SAM and HAA reaction from the substrate (**1**). The TDP group of the substrate is not shown. Key distances are given in angstroms.

Molecular dynamics (MD) simulations and QM/MM computations were performed to evaluate the energetics of DesII‐catalyzed deamination of **1** based on the structure of the binary DesII·SAM complex. The substrate (**1**) was found to dock into the active site in several different conformations with similar energy minima (Figure S4, Supporting Information), one of which was a twist‐boat conformation in which the C3′‐OH and ionized C4′‐NH_3_^+^ groups can both form H‐bonds with E408 (Figure S5, Supporting Information). Reduction of SAM by the reduced [Fe_4_S_4_]^+^ cluster is coupled to reductive cleavage of the C─S bond to afford a 5′‐deoxyadenosyl radical (5′dA•) with a barrier of 15.6 kcal mol^−1^ (**RC** → **IC1**, **Figure** **4**). QM/MM optimization of the **IC1** complex places the bound 5′dA• radical in a local energy minimum of 5.8 kcal mol^−1^ higher than the initial Michaelis complex (**RC**). The C5′‐methyl radical in **IC1** is 2.55 Å from the C3′ H‐atom of **1**, indicating ideal positioning for H‐abstraction. The computed barrier for the generation of 5′‐dA• and the following H‐atom abstraction （HAA） from C3′ of **1** is 15.6 kcal mol^−1^ (**RC** → **TS1**) and 13.4 kcal mol^−1^ (**IC1** → **TS2**), respectively.

**Figure 5:**
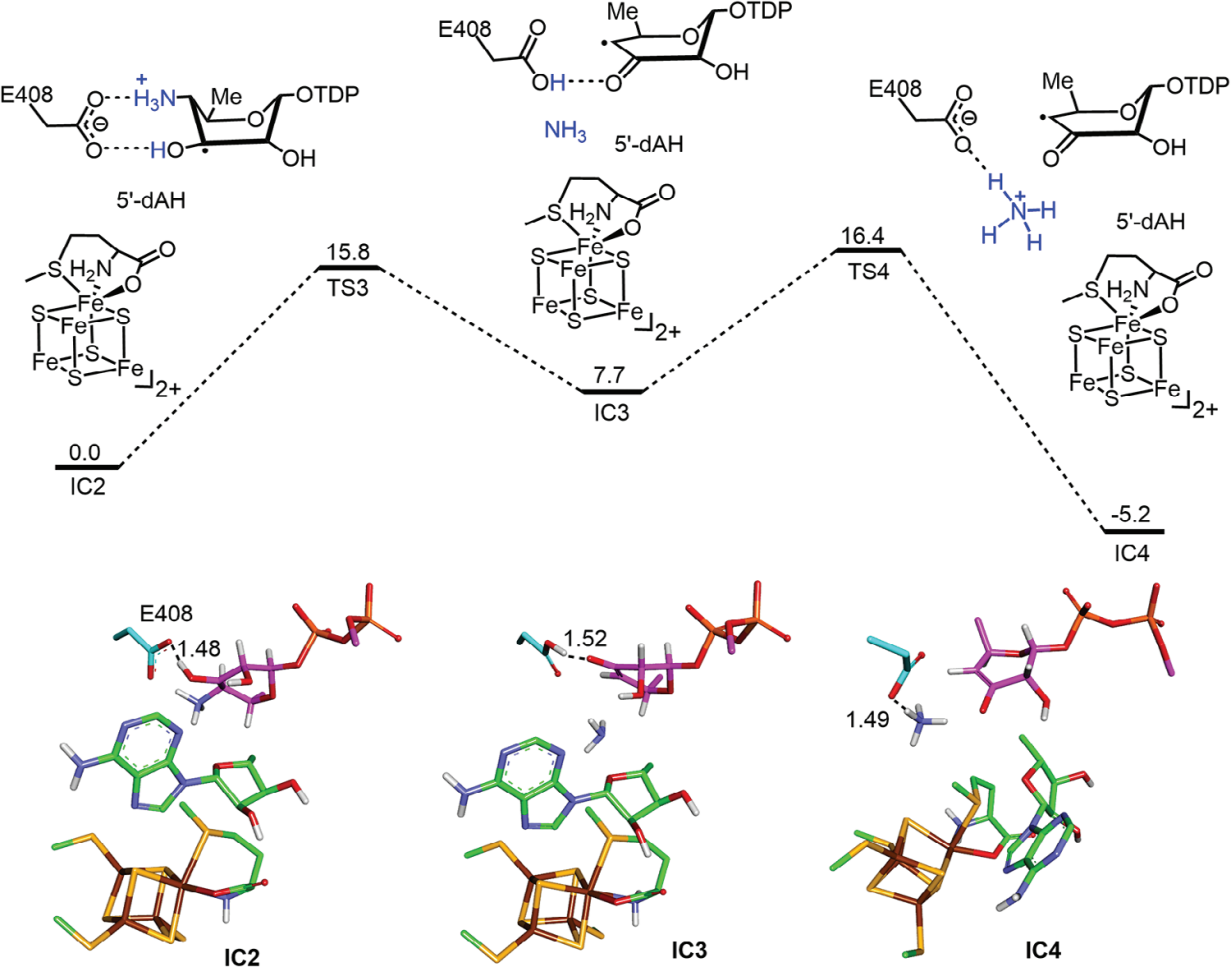
QM(TPSSh/def2‐TZVP)/MM calculated energy profile (kcal mol^−1^) for deamination from the boat conformation of the substrate radical. The TDP group of the substrate is not shown. Key distances are given in angstroms.

Following H‐atom abstraction, the substrate radical can relax from an initial twist boat conformation to two or more stable conformations identified via QM/MM metadynamics (Figure S6, Supporting Information). These include a boat (**IC2**) and a chair (**IC2′**) conformation with similar energies. In the boat conformation, the protonated C4′‐amino group and C3′‐hydroxyl maintain both H‐bonds with the two oxygen centers of the E408 carboxylate. In the chair conformation, however, both functional groups are H‐bonded to the same oxygen center of the E408 carboxylate (**Figure** **5**; Figure S6, Supporting Information). Moreover, the boat form with both substituents at C4′ and C3′ in diequitorial orientation appears to be 14.8 kcal mol^−1^ more stable than the diaxial counterpart (Figure S7, Supporting Information). The minimum energy pathway (Figure 5) beginning from the boat conformation (**IC2**) results in dissociation of the amino group coupled with proton transfer from the C3′ hydroxyl to E408 with a barrier of 15.8 kcal mol^−1^ (**IC2 → TS3**). This is followed by proton transfer from E408 to ammonia with a barrier of 8.7 kcal mol^−1^ (**IC3 → TS4**). In contrast, deamination from the chair conformer did not afford a stable enol radical (**IC3′**) for the subsequent reactions (Figure S8, Supporting Information).

The minimum energy deamination pathway is consistent with previous experimental results, implying direct elimination of ammonia from the substrate radical (Figure 1B, pathway **b**).^[^ ^14^, ^15^ ^]^ Moreover, if no other steps following the formation of **IC4** (i.e., reduction, product dissociation, etc.) strongly limit turnover at saturation, then the calculated *k_cat_* value is 3.0 min^−1^ at 25 °C, which compares favorably with experiment.^[^ ^2^ ^]^ Nevertheless, we also examined the alternative mechanism involving deamination via radical‐mediated 1,2‐migration of the amino group to generate a carbinolamine radical intermediate (Figure 1B, pathway **a**) computationally. Transition states for 1,2‐migration of the protonated amino group indeed could be located from both the boat (**IC2**) and chair conformations (**IC2′**); however, both of these transition states had energies of 10 kcal mol^−1^ or greater than that for direct elimination (Figure S9A,B, Supporting Information). Consequently, the combined computational and experimental results favor deamination via direct elimination without the intermediacy of a carbinolamine radical (**7**).

A direct elimination mechanism was also considered in which the *α*‐hydroxyalkyl radical at C3′ protonates the ammonia leaving group concerted with its elimination. In this case, a high energy barrier of ca. 32 kcal mol^−^^1^ was observed (Figure S9C, Supporting Information), suggesting the importance of E408 or another H‐bond acceptor in binding the substrate and mediating the proton transfer events. However, DesII‐E408A and E408Q mutants were both constructed and found to retain deaminase activity comparable to that of wildtype enzyme as well as the ability to catalyze the dehydrogenation of TDP‐d‐quinovose (Section S3 and Figures S18 and S19, Supporting Information). This implies that another proton acceptor such as a water molecule or another active site residue, may be able to serve in the same capacity as E408 (i.e., a proton shuttle) after the latter has been removed via mutation. Inspection of the active site showed that the residue D456 sits adjacent to E408 (Figure S10, Supporting Information) and may play such a role as the DesII‐D456A mutant was found to be catalytically inactive (Section S3 and Figures S18 and S19, Supporting Information). Moreover, computations suggested that mutation of E408 to alanine allowed D456 to directly engage in similar H‐bonding interactions with the substrate instead (Figure S11, Supporting Information). Consequently, deamination of the α‐hydroxyalkyl radical from the boat conformation in the DesII‐E408A active site was computed to proceed with a similar activation energy equal to 16.6 kcal mol^−1^ (Figure S11, Supporting Information).

### 2.3 Deamination Versus Dehydrogenation

**Figure 6:**
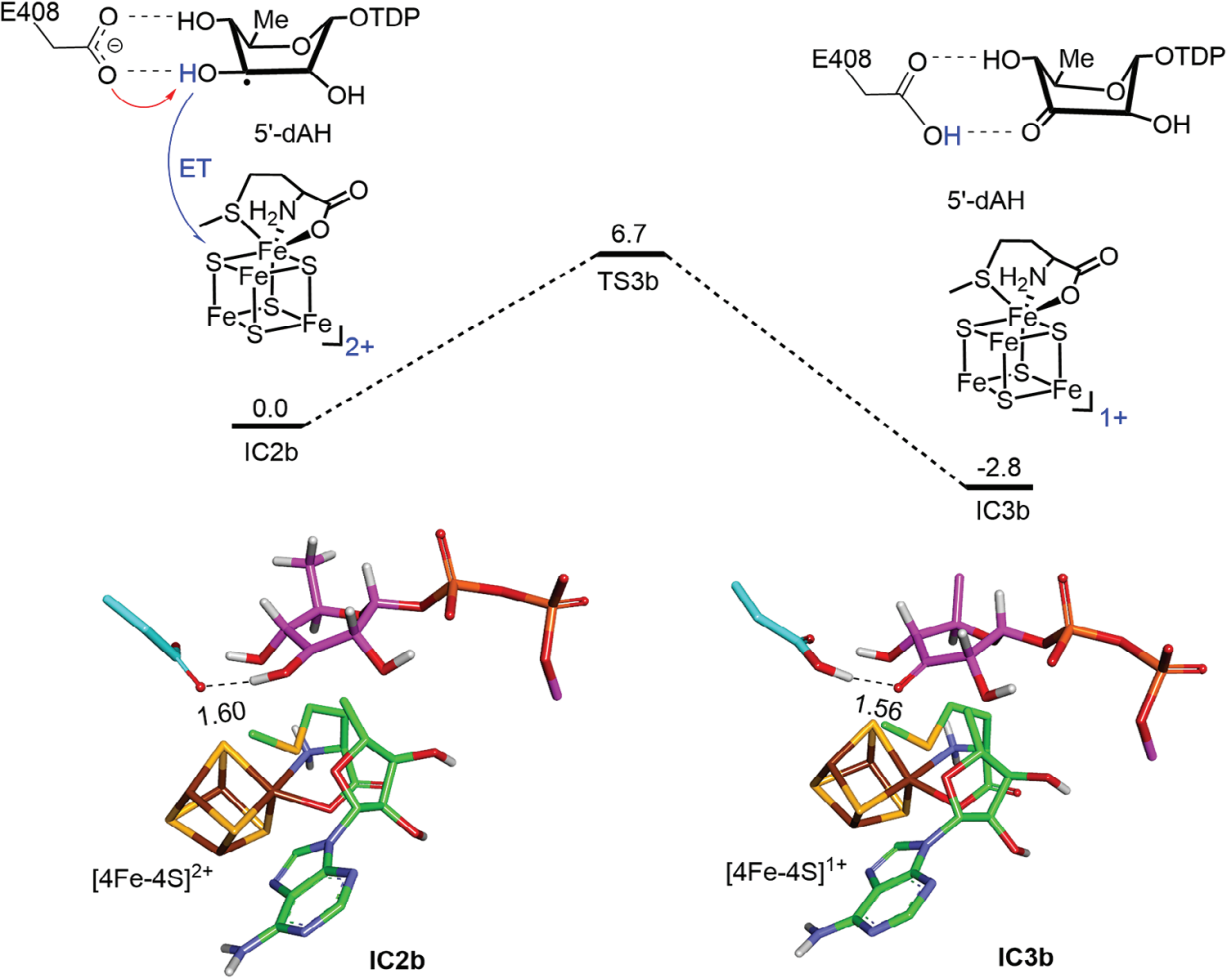
QM(TPSSh/def2‐TZVP)/MM calculated energy profile (kcal mol^−1^) for oxidation of the C3′ radical species via a PCET mechanism. The TDP group of the substrate is not shown. Key distances are given in angstroms.

The DesII‐catalyzed dehydrogenation of TDP‐d‐quinovose (**4**) was also studied computationally based on the binary DesII·SAM structure. The substrate was again found to dock into the DesII active site with SAM in a twist‐boat conformation similar to that of **1**, and E408 again formed H‐bonds with both the C3′ and C4′ hydroxyl groups (Figure S12, Supporting Information). Generation of the corresponding C3′ substrate radical followed a similar energy profile (Figure S13, Supporting Information) to yield the substrate radical **10** in a stable boat conformation where the H‐bonds with E408 are maintained (**Figure** **6**). This configuration had a geometry consistent with that reported from EPR investigations (Figure S14, Supporting Information).^[^ ^24^ ^]^ Starting from the C3′ radical intermediate, the most favorable pathway involves deprotonation of the *α*‐hydroxyalkyl radical at C3′ by E408 concomitant with electron transfer to the [Fe_4_S_4_]^2+^ complex (Figure 6). This proton‐coupled electron transfer reaction is characterized by a 6.7 kcal mol^−1^ barrier to afford the dehydrogenation product **5**. In contrast, dehydration of **10** involved a much higher barrier of 23 kcal mol^−1^ compared to dehydrogenation and the corresponding deamination reaction (Figure S15, Supporting Information). Hence, **5** is the only product observed when **4** is incubated with DesII.

To get some insight into the divergent reactivity of substrates **1** and **4**, the energy penalties associated with oxidation of the C3′‐ketyl radicals in the twist‐boat conformation were compared when C4′ carried a protonated amine versus a hydroxyl group. As illustrated in Figure S16 (Supporting Information), removing an electron from the latter radical has a much lower energy penalty than the former. This may explain why the biosynthetic substrate carrying a C4′‐amino group (**1**) undergoes deamination while TDP‐quinovose (**4**) is instead oxidized. Unlike the hydroxyl group, the positively charged amino group may inhibit electron transfer from the initially formed substrate radical.

## 3 Conclusion

Both experimental and computational results suggest that DesII catalyzes the deamination of **1** via direct elimination without a carbinolamine intermediate (Figure S17, Supporting Information). This contrasts the currently accepted mechanism of EAL and implies that radical‐mediated lyase activity need not be restricted to a single mechanistic paradigm. Nevertheless, as more and more deaminases and dehydratases are characterized, direct elimination as opposed to migration is the predominant mechanism with the latter primarily restricted to the AdoCbl‐dependent enzymes.^[^ ^23^ ^]^ Why this might be the case is unclear; however, it may be related to the shared chemistry of the AdoCbl‐dependent family of enzymes that includes both lyases and mutases, the latter of which necessarily catalyze group migration reactions.^[^ ^32^, ^33^ ^]^ Moreover, a more stringently organized active site and constrained substrate conformation may be required to effect migration instead of direct elimination. This may also help to prevent off‐pathway side reactions and ensure regeneration of the AdoCbl cofactor.

In contrast, the radical SAM enzymes are much more mechanistically diverse, with over half a million protein sequences annotated and an ever‐increasing number of reactions reported to be catalyzed.^[^ ^6^, ^34^, ^35^ ^]^ The radical SAM enzymes are also expected to exert tight control over their radical intermediates to prevent facile side reactions, but they seem particularly susceptible to such alternative reaction outcomes.^[^ ^36^ ^]^ DesII is a case in point, which can be converted to a dehydrogenase by simply swapping one leaving group for another. Consequently, even minor perturbations in the substrate or active site structure can result in new chemical reactions and potentially the ready evolution of new biosynthetic enzymes.^[^ ^37^, ^38^ ^]^ This may help explain the radical SAM enzymes’ broad mechanistic diversity and versatility.

## Conflict of Interest

The authors declare no conflict of interest.

## Supporting information

Supporting Information

## Data Availability

The data that support the findings of this study are available from the corresponding author upon reasonable request.
