# Supplementary material for: Mechanistic Insights from the Crystal Structure and Computational Analysis of the Radical SAM Deaminase DesII
Source: Adv Sci (Weinh). 2024 Jun 28;11(33):2403494. doi: 10.1002/advs.202403494 (PMC11434129; doi:10.1002/advs.202403494)
Supplement: Supplementary file 1 — Supporting Information [file ADVS-11-2403494-s001.pdf]

ADVANCED SCIENCE
Open Access

Supporting Information

for *Adv. Sci.*, DOI 10.1002/advs.202403494

Mechanistic Insights from the Crystal Structure and Computational Analysis of the Radical SAM Deaminase DesII

Xueli Hou, Jianqiang Feng, Joseph Livy Franklin, Ryan Russo, Zhiyong Guo, Jiahai Zhou, Jin-Ming Gao*, Hung-wen Liu* and Binju Wang**

Supporting Information

Mechanistic Insights from the Crystal Structure and Computational Analysis of the Radical SAM Deaminase DesII

Xueli Hou+,[a,b] Jianqiang Feng+,[c] Joseph Livy Franklin,[d] Ryan Russo,[d] Zhiyong Guo,[e] Jiahai Zhou,*[b,f] Jin-Ming Gao,*[a] Hung-wen Liu,*[d,g] and Binju Wang*[c]

Table of Contents

S1 X-ray crystallography ... 3
S1.1 Cloning of the *N*-terminal His_8-tagged *desII* gene ... 3
S1.2 Expression of DesII ... 3
S1.3 Anaerobic purification of DesII ... 3
S1.4 Crystallization of DesII ... 3
S1.5 Data collection and structure determination ... 3
S1.6 Structural analysis ... 3
S2 Computational methods ... 4
S2.1 System setup ... 4
S2.2 MD simulations ... 4
S2.3 QM/MM MD simulations ... 4
S2.4 QM/MM metadynamics simulations of sugar puckering ... 4
S2.5 QM/MM simulations ... 4
S3 DesII mutants ... 5
S3.1 Preparation of mutant enzymes ... 5
S3.2 Reconstitution ... 5
S3.3 Assays ... 5
Supplementary Tables ... 7
Supplementary Figures ... 8
References ... 18

S1 X-ray crystallography

S1.1 Cloning of the *N*-terminal His_8-tagged *desII* gene

The *desII* gene was amplified by PCR from the plasmid pDesIIWt, which was previously derived from pET-24b (+). The forward amplification primer (5′-CGC GGA TCC ATG ACC GCC CCC GCC CTT TCC GCC-3′) contained a BamHI restriction site. The reverse amplification primer (5′-CCG CTC GAG **TCA** GCG CAG GAA GCC GCG GGC CTC-3′) contained an XhoI restriction site and a stop codon (underlined and bold, respectively). The PCR product was isolated and digested with BamHI and XhoI using standard procedures. [1] The digested product was inserted into the pSJ5 expression vector containing the TrxA fusion partner, His_8-tag and TEV site that had been similarly digested. The resulting plasmid construct was confirmed by DNA sequencing at Tsingke Biotechnology Co., Ltd. and designated pNterm-DesIIWt.

S1.2 Expression of DesII

Both the *C*-terminal and *N*-terminal His-tagged constructs of *desII* were overexpressed in *E. coli* BL21(DE3) containing plasmid pDB1282 or pDB1281 as previously described. [2] The selenomethionine-derivatized (Se-Met) DesII was produced by *E. coli* BL21(DE3) containing the pDB1282 plasmid with expression in M9 media supplemented with SeMet, seven other amino acids (isoleucine, leucine, valine, lysine, threonine, phenylalanine, cysteine), L-(+)-arabinose, $FeCl_3$ and IPTG as previously described. [3]

S1.3 Anaerobic purification of DesII

DesII purification was performed under anaerobic conditions unless otherwise specified. Cells from a ten liter culture were suspended with 100 mL of 25 mM Tris HCl buffer (pH 8.0) containing 300 mM NaCl, 5 mM β-mercaptoethanol, 1 mM phenylmethylsulfonyl fluoride (PMSF), 1 µg/mL benzonase nuclease and 1 mg/L lysozyme. Cells were lysed by sonication (5 s on and 10 s off, for ca. 30 min), and the cell debris was removed by centrifugation at 18000 rpm and 4 °C for 30 min. The supernatant was loaded onto a column with 15 mL Ni-NTA resin (GE Healthcare), and the His-tagged protein was eluted with 25 mM Tris HCl buffer (pH 8.0) containing 300 mM NaCl, 300 mM imidazole and 5 mM β-mercaptoethanol. The isolated protein was concentrated using Amicon ultra filtration units (Millipore) with a 30 kDa MWCO. The concentrated *C*-terminal His_6-tagged DesII construct was further purified by ion-exchange chromatography on a HiTrapTM 26/10 QFF column (GE Healthcare). The protein was eluted with 25 mM Tris HCl buffer (pH 8.0) containing 300 mM NaCl and 4 mM DTT. The concentrated *N*-terminal His_8-tagged DesII construct was treated with the TEV enzyme to remove the TrxA fusion partner and His-tag. The concentrated protein concentration was then determined with a Thermo Scientific Nanodrop.

S1.4 Crystallization of DesII

The DesII·SAM binary complex was crystallized by adding five-fold SAM to a solution of 12 mg/mL purified *C*-terminal His_6-tagged constructs of DesII obtained as described in Section S1.3 and incubated on ice for 30 min. The protein was then combined with the reservoir solution at a 1:1 ratio in a 2 µL volume and equilibrated at 20 °C against 50 µL reservoir solution by the sitting drop vapor diffusion method. Crystals of the DesII complex appeared after 14 days at 20 °C in 0.1 M Tris pH 8.5, 16–30% PEG4000 and 0.2 M lithium sulfate. In order to improve the quality of the crystals, the crystallization conditions were optimized by changing the pH of the buffer solution and the concentration of the precipitant. The crystals were grown in 0.1 M Tris pH 8.5, 32% PEG4000 and 0.2 M lithium sulfate under anaerobic conditions. Crystals of DesII without substrate or SAM bound were grown in 0.1 M MES monohydrate pH 6.0 containing 20% PEG 6000 and 1.0 M LiCl. Crystals were flash-frozen in liquid nitrogen after being dipped into a solution containing 5% glycerol.

S1.5 Data collection and structure determination

X-ray diffraction data of DesII were collected at a wavelength of 0.9785 Å at beamline 19U1 of the Shanghai Synchrotron Radiation Facility (SSRF). The data sets were indexed, integrated and scaled using the Porpoise XDS [4] or DIALS [5] packages before being merged by aimless in ccp4i. [6] Both crystals belonged to space group $P2_12_12_1$, and the statistics for data collection are summarized in Table S1. The structure of SeMet·DesII in complex with SAM was determined by single-wavelength anomalous dispersion (SAD). A total of 31 selenium sites were identified in one asymmetric unit by Autosol and used for model building with Autobuild in the PHENIX package. [7] Using the atomic coordinates of the SeMet·DesII·SAM complex as the search model, the structure of the DesII·SAM complex was determined by molecular replacement using Phaser. Iterative cycles of model rebuilding and refinement were carried out using COOT, [8] and PHENIX [7]. The structure of DesII without substrate or SAM bound was determined by molecular replacement using PHASER and the atomic coordinates of the DesII·SAM complex as the search model. PROCHECK [9] and MolProbity [10] were used to assess the overall quality of the structural models. Refinement statistics for each final model are summarized in Table S1. All structure figures were prepared using PyMol 1.3 (Schrödinger, LLC) [11] unless specified otherwise.

S1.6 Structural analysis

A search for structures similar to that of DesII in the Protein Data Bank was performed using the program DALI, [12] and the two radical SAM enzymes BlsE (PDB 7VOB) and MoaA (PDB 1TV8) were identified as having the greatest structural similarity. BlsE catalyzes the radical mediated 1,2-diol dehydration of cytosylglucuronic acid during the biosynthesis of blasticidin S. [2] In contrast, MoaA catalyzes the conversion of GTP to precursor Z, which involves a radical-mediated intramolecular rearrangement of its substrate. [13-15] BlsE had the highest Z score (18.3) and a root-mean-square deviation (rmsd) of 3.7 Å for 328 residues, and MoaA had the second highest Z score (17.8) and a rmsd of 3.4 Å for 328 residues.

The structures of BlsE, MoaA and DesII show a common structural core involved in the RS domain binding the [Fe_4S_4] cluster and SAM (Figures S1). In terms of the structural differences, DesII has a single iron-sulfur cluster, whereas both BlsE and MoaA are twitch radical SAM enzymes and thus bind an auxiliary iron-sulfur cluster near the *C*-terminus. [16, 17] The greatest similarity in their sequence alignments was also concentrated in the RS domain (Figures S2). The *N*-terminal domain of DesII, which is characterized by six α-helices, was searched separately with DALI, and transcription factor PF0095 (PDB 2QLZ) from *Pyrococcus furiosus* was returned with the highest Z score (4.8) and a rmsd of 2.6 Å for 220 residues. However, inspection of both the structures (Figure S3) as well as the sequence alignments (ca. 20% identity) does not suggest any evidence of homology between these protein sequences.

S2 Computational methods

S2.1 System setup

The initial structure was prepared based on the DesII·SAM complex structure (PDB 8HZV). The protonation states of the titratable residues (His, Glu, Asp) were assigned based on pK_a values from the PROPKA software [18] in combination with careful visual inspection of local hydrogen-bond networks. Histidine residues His50 and His146 were protonated at the δ position, but His17, His27, His71, His203, His207, His235, His359 and His390 were protonated at the ε position. All glutamic acid and aspartic acid residues were deprotonated. The force field of the [Fe_4S_4]-SAM complex was parameterized using the "MCPB.py" modeling tool of AmberTools18. [19, 20] The Amber ff14SB force field [21] was employed for the protein residues. The general AMBER GAFF force field [22] was used for substrates, while partial atomic charges were obtained from the RESP method [23] using the B3LYP/6-31G* level of theory. Sodium ions were added to the protein surface to neutralize the total charge of the system. The resulting three systems were then solvated in a rectangular box of TIP3P waters extending up to a minimum distance of 16 Å from the protein surface.

S2.2 MD simulations

The whole system was fully minimized using the combined steepest descent and conjugate gradient method. The system was then gently annealed from 10 to 300 K under canonical ensemble for 50 ps with a weak restraint of 25 kcal/mol/Å on the protein. To achieve a uniform density after heating dynamics, 1 ns of density equilibration was performed under the NPT ensemble at the target temperature of 300 K and target pressure of 1.0 atm. Afterwards, all constraints on the protein were removed and the system was further equilibrated for 2 ns under the NPT ensemble to well-settle the pressure and temperature. Finally, a productive MD simulation under the NPT ensemble was conducted for 100 ns for the enzyme system. During all MD simulations, covalent bonds involving hydrogen were constrained using SHAKE, and an integration step of 2 fs was used. All MD simulations were performed with the GPU version of the Amber 18 package.

S2.3 QM/MM MD simulations

One representative snapshot extracted from the converged MD trajectory was used for the subsequent QM/MM MD simulations. All QM/MM Born-Oppenheimer MD simulations were performed with the CP2K 5.0 package, combining the QM program QUICKSTEPS [24] and the MM driver FIST. In this approach, a real space multigrid technique is used to compute the electrostatic coupling between the QM and MM regions. [25, 26] The QM region was treated at the DFT (B3LYP) level, employing the dual basis set of Gaussian and plane-waves (GPW) formalism, whereas the remaining part of the system was treated at the classical level using the same parameters as in the classical MD simulations. The Gaussian double-ζ valence polarized (DZVP) basis set was used to expand the wave function, [27] while the auxiliary plane-wave basis set with a density cutoff of 360 Ry and GTH pseudopotentials [28] was utilized to converge the electron density. Dangling bonds between the QM and MM regions were capped with hydrogen atoms. To speed up the calculation of Hartree-Fock exchange within B3LYP, the auxiliary density matrix method (ADMM) was used. [29] All QM/MM MD simulations were performed under the NVT ensemble using an integration time step of 0.5 fs, the system was equilibrated without any constraint for 2 ps.

S2.4 QM/MM metadynamics simulations of sugar puckering

The conformational free energy landscape (FEL) of the 4-amino-4,6-dideoxy-D-glucose ring of the substrate (**1**) bound to DesII was computed by QM/MM metadynamics simulations. The simulations were initiated from a snapshot of the QM/MM MD simulations. The QM/MM metadynamics were performed by the interface of AmberTools22 [30] and ORCA [31] v5.02 with an integration time step of 0.5 fs, and the metadynamics algorithm was provide by the PLUMED2 [32] plugin. The 4-amino-4,6-dideoxy-D-glucose ring unit was included the QM region, which was treated by semiempirical extended tight-binding method GFN2-xTB [33] v6.4.1. The Cremer-Pople puckering coordinates [34] theta and phi (θ, ϕ) of the pyranose ring were used as collective variables. The height and the width of the Gaussian terms were set at 0.6 kcal/mol and 0.1 rad, respectively, and the deposition time was 50 MD steps (25 fs). The simulation stops once no quantitative change in FEL is observed. The conformational FEL of both substrate and its radical intermediate are shown in Figure S4 and Figure S6.

S2.5 QM/MM simulations

All QM/MM calculations were performed using ChemShell [35, 36] combining Turbomole [37] for the QM region and DL_POLY [38] for the MM region. The electronic embedding scheme [39] was used to account for the polarizing effect of the enzyme environment on the QM region. Hydrogen link atoms with the charge-shift model were applied to treat the QM/MM boundary. During QM/MM geometry optimizations, the QM region was studied with the TPSSh density functional with two levels of theory. For geometry optimization, the

double-ζ basis set def2-SVP, collectively labeled as B1, was used. The energies were further corrected with the larger basis set def2-TZVP for all atoms, labeled as B2. Dispersion corrections computed with Grimme's D3 method [40-42] were included in all QM calculations. For the study of SAM activation and hydrogen atom transfer, the QM region included the [Fe_4S_4] cluster, SAM, substrate and the side chains of Cys141, Cys145, Cys148. In addition to the above, Glu408 is also added to the QM region for the study of deamination and the PCET process. For the case of E408A mutant, Asp456 is added to the QM region.

S3 DesII mutants

S3.1 Preparation of mutant enzymes

Site directed mutagenesis was performed with the PCR primers shown below using KOD Hot Start DNA Polymerase and *C*-terminal His-tagged DesII in a pET24b(+) vector as a template. [43] The PCR products were ligated using NEBuilder HiFi assembly mix. Sanger sequencing of the mutants was performed by Eton Biosciences. The mutant and wild type proteins were heterologously expressed in *E. coli* (BL21 Star) and isolated aerobically via Ni-NTA solid phase extraction as previously described [44] and dialyzed against 50 mM sodium phosphate (pH 8.0, NaOH) containing 15% glycerol. The resulting protein was further purified aerobically using anion exchange FLPC with a Mono-Q 16/10 column (20 mL volume) at a flow rate of 5 mL/min with solvents A 50 mM sodium phosphate (pH 7.5, NaOH) and B 50 mM sodium phosphate 500 mM NaCl (pH 7.5, NaOH). The elution gradient was 0% B for 20 mL, 0% to 50% B in 100 mL, 50% to 70% B in 100 mL, 70% to 100% B in 20 mL, 100% B for 40 mL, return to 0% B in 60 mL. DesII typically elutes after approximately 150 mL. Aliquots containing DesII were pooled, dialyzed against 50 mM sodium phosphate (pH 8.0, NaOH) containing 15% glycerol before concentration via centrifugal filtration, flash freezing in liquid N_2 and storage at –80 °C prior to use.

E408A Mutation primers
Forward-ACGTGTACCTGTACCGCGCTGCCGGCTTCCCCGACCTGGA
Reverse-CCGGCAGCGCGGTACAGGTACACGTCGC

E408Q Mutation primers
Forward-ACGTGTACCTGTACCGCCAGGCCGGCTTCCCCGACCTGGA
Reverse-CCGGCCTGGCGGTACAGGTACACGTCGC

D456A Mutation primers
Forward-CTTCATGGCTGGCTTCGATCAGGTCGTCAC
Reverse-CGAAGCCAGCCATGAAGTACTCGTCGCCG

Wildtype DesII does not express well yielding less than 5 mg DesII protein per 6 L culture and brown-tinted solutions at each step in the aerobic purification procedure. The DesII-D456A mutant expresses poorly with less than 1 mg DesII protein per 6 L culture and faintly brown-tinted solutions during purification. The DesII-E408A and DesII-E408Q mutants express extremely poorly with less than 0.25 mg DesII protein per 6 L culture and neither colored solutions nor readily identifiable peaks observable by FPLC. Nevertheless, partial purification was possible despite significant contamination with a 75 kD protein from the *E. coli* expression system, which has a similar FPLC elution time. An SDS-PAGE of the four different constructs following reconstitution (see below) is shown in Figure S17.

S3.2 Reconstitution

All four DesII constructs were reconstituted the same way. A 500 µL solution containing 0.1–0.2 mg of a DesII construct was dialyzed at 4 °C overnight against 1 L 25 mM EPPS buffer (pH 8.0, NaOH) and 10% glycerol under a N_2 atmosphere. The next morning, the dialysis system was brought into a Coy anaerobic chamber (98% N_2, balance H_2), and the protein solution transferred to a 1 mL conical vial on a cold block (ca. 10 °C) where it was allowed to equilibrate with the anaerobic atmosphere for at least 30 min with periodic mixing before 25 µL 100 mM DTT was added making the solution 5 mM in DTT. The solution was allowed to incubate for another 30 min with periodic mixing before 20 µL 40 mM $Fe(NH_4)_2(SO_4)_2$ in H_2O was added over 10 min (i.e., 2 µL/min). This is then immediately followed by the addition of 20 µL 40 mM Na_2S in 25 mM EPPS buffer (pH 8.0, NaOH) containing 1 mM DTT over 20 min (i.e., 1 µL/min). The resulting solution containing 0.2–0.4 mg/mL DesII construct, 5 mM DTT, 1.6 mM $Fe(NH_4)_2(SO_4)_2$, 1.6 mM Na_2S, 10% glycerol and 25 mM EPPS (pH 8.0) had a distinctly tan-brown color that darkened to a brown-olive-green color over next 2 h. A similar color change was observed in the case of all four DesII constructs. The clear, colored solution was then applied to a 10 mL Sephadex G25 size exclusion column previously equilibrated with 25 mM EPPS buffer (pH 8.0, NaOH) containing 1 mM DTT. The protein eluted in brown-olive-green colored clear solutions that are positive for protein via spot test with Bradford reagent. These aliquots were pooled and concentrated via centrifugal filtration to a final volume of ca. 500 µL, and the protein concentration was determined by Nanodrop. SDS-PAGE of the final reconstituted DesII-constructs is shown in Figure S17, and indicate that the DesII-E408A and DesII-E408Q mutants are no more than 20% pure (see above discussion). The enzyme preparations were stored at ca. 10 °C in the anaerobic chamber prior to assay.

S3.3 Assays

The TDP-sugar substrates (i.e., **1** & **4**) used were in the form of their ammonium salts and prepared as previously described. [44, 45] Activity assays of the DesII mutants were performed in 50 µL reactions containing 25 mM EPPS buffer (pH 8.0, NaOH), 1 mM DTT,

500 µM SAM, 150 µM TDP-4-amino-4,6-dideoxy-D-glucose (**1**), 1 µM DesII (assuming 20% purity in the case of the E408 mutants) and 4 mM $Na_2S_2O_4$ added last to initiate the reaction. Each mutant was assayed in parallel with DesII wildtype as a positive control as well as a negative control with no enzyme. Reactions were incubated for 10 min before removal from the anaerobic chamber, centrifugal filtration to remove protein and freezing before HPLC analysis. HPLC analysis utilized a Dionex CarboPac PA1 column (4 × 250 mm) with H_2O as solvent A and 1 M NH_4AcO in water as solvent B. The HPLC elution gradient was isocratic 4% B from 0 to 2 min, linear 4% to 22% B over 20 min, linear 22% to 30% B over 20 min, linear 30% to 4% B over 5 min and finally isocratic 4% B for 3 min. The substrates **1** and SAM typically elute at ca. 12.0 and 18.0 min respectively. The deaminated product **2** elutes at ca. 32.7 min, whereas 5′-deoxyadenosine elutes in the void volume (see Figure S18). Detection was by UV absorbance at 267 nm.

Extent of reaction was determined from the relative peak integrations of the deaminated product versus residual substrate. No turnover was observed in the presence of the DesII-D456A mutant at 10 or 20 min (Figure S18A). Longer incubations of over 60 min also showed no product formation. In the presence of no more than 1 µM DesII-E408A mutant, 11% deamination was observed at 10 min for an initial rate of ca. 1.6 µM/min versus 65% in the presence of 1 µM DesII-wildtype (positive control) for an initial rate of ca. 9.8 µM/min (Figure S18B). In the presence of no more than 1 µM DesII-E408Q mutant, 22% deamination was observed at 10 min for an initial rate of ca. 3.4 µM/min versus 28% deamination in the presence of 1 uM DesII-wildtype (positive control) for an initial rate of ca. 4.2 µM/min (Figure S18C). Therefore, the DesII-D456A mutant is catalytically inactive whereas the DesII-E408A and DesII-E408Q mutants have activity comparable to that of wildtype.

The DesII-E408A and DesII-E408Q catalyzed reactions were also analyzed by LC/MS. Reactions were performed anaerobically in 50 mM HEPES buffer (pH 8.0) with 200 mM NaCl, 10% glycerol, 1 mM SAM, 1 mM dithionite and 1 mM TDP-substrate. DesII was added last to a final concentration of 0.15 mg/mL total protein in a total volume of 50 µL. The reactions were incubated anaerobically for 1.0–2.5 h, removed from the anaerobic chamber, diluted to 450 µL with water and deproteinized via centrifugal filtration with YM-10 filters prior to analysis. Liquid-chromatography mass spectrometry (LCMS) analysis was performed using an Agilent Technologies 1260 Infinity HPLC system equipped with a 6230 ToF mass spectrometer with electrospray ionization. Column elution utilized the solvents 0.1% formic acid in water (A) versus acetonitrile (B) according to the following program: isocratic 1% B from 0 to 5 min, linear 1% to 20% B over 5 min, linear 20% to 30% B over 3 min, linear 30% to 75% B over 3 min, linear 75% to 85% B over 3 min followed by linear return from 85% to 1% B in 0.5 min.

For each assay involving one of the three enzyme constructs (wildtype, E408A or E408Q) with the deamination substrate TDP-4-amino-4,6-dideoxy-D-glucose (**1**), extracted ion chromatogram (EIC) peaks for *m*/*z* consistent with the protonated substrate **1** ion or protonated deamination product **2** ion (both carbonyl and hydrate) were observed at 4.9 and 13.2 min, respectively. The ratio of the total product EIC peak integration to the sum of the product and residual starting material EIC integrations was >95% for DesII-wildtype, 50% for the DesII-E408A mutant and 60% for the DesII-E408Q mutant. The same analysis was performed with the dehydrogenation substrate TDP-D-quinovose (**4**), and EIC peaks for *m*/*z* consistent with the protonated substrate **4** ion and protonated dehydrogenation product **5** ion (both carbonyl and hydrate) were all observed at 12.2 min. The ratio of the total product EIC peak integration to the sum of the product and residual substrate EIC integrations was 90% for wildtype DesII, 50% for the DesII-E408A mutant and 80% for the DesII-E408Q mutant.

Supplementary Tables

Table S1. Data collection and refinement statistics.

Dataset	SeMet-DesII*	DesII •SAM	apo-DesII
PDB ID		8HZV	8HZY
**Data collection**			
Wavelength (Å)	0.9785	0.9785	0.9785
Space group	$P2_12_12_1$	$P12_11$	$P2_122_1$
**Cell dimensions**			
a, *b*, *c* (Å)	67.71, 109.8, 136.42	69.19, 140.8, 116.0	97.10, 105.2, 118.2
α, *β*, *γ* (°)	90.0, 90.0, 90.0	90.0, 96.3, 90.0	90.0, 90.0, 90.0
Resolution (Å) †	48.05-2.10 (2.16-2.10)	49.19-2.33 (2.37-2.33)	32.98-2.04 (2.08-2.04)
*R*merge†	0.050 (0.925)	0.097 (0.989)	0.198 (0.661)
I/σ*I* †	18.1 (2.1)	16.8 (2.2)	5.8 (2.1)
Completeness (%)†	97.5 (100.0)	99.9 (100.0)	100.0 (100.0)
Redundancy†	5.8 (6.1)	6.8 (6.7)	6.2 (6.0)
CC1/2†	0.999 (0.744)	0.998 (0.686)	0.990 (0.680)
**Refinement**			
Resolution (Å)		49.19-2.33 (2.41-2.33)	32.98-2.04 (2.11-2.04)
No. reflections†		94068 (9412)	77577 (7672)
R_{work}/ R_{free}		0.2046/0.2358	0.1987/0.2363
No. atoms		15473	7926
Protein		14871	7133
Ligand/ion		73	16
Water		529	777
***B*-factors**			
Protein		46.9	23.1
Ligand/ion		44.6	33.8
Water		42.8	31.4
**R.M.S deviations**			
Bond lengths (Å)		0.0031	0.0095
Bond angles (º)		0.60	1.00
**Ramachandran plot**			
favored (%)		97.20	97.25
allowed (%)		2.59	2.53
outlier (%)		0.21	0.22

*Data were scaled anomalously.

†Highest resolution shell is shown in parentheses.

Supplementary Figures

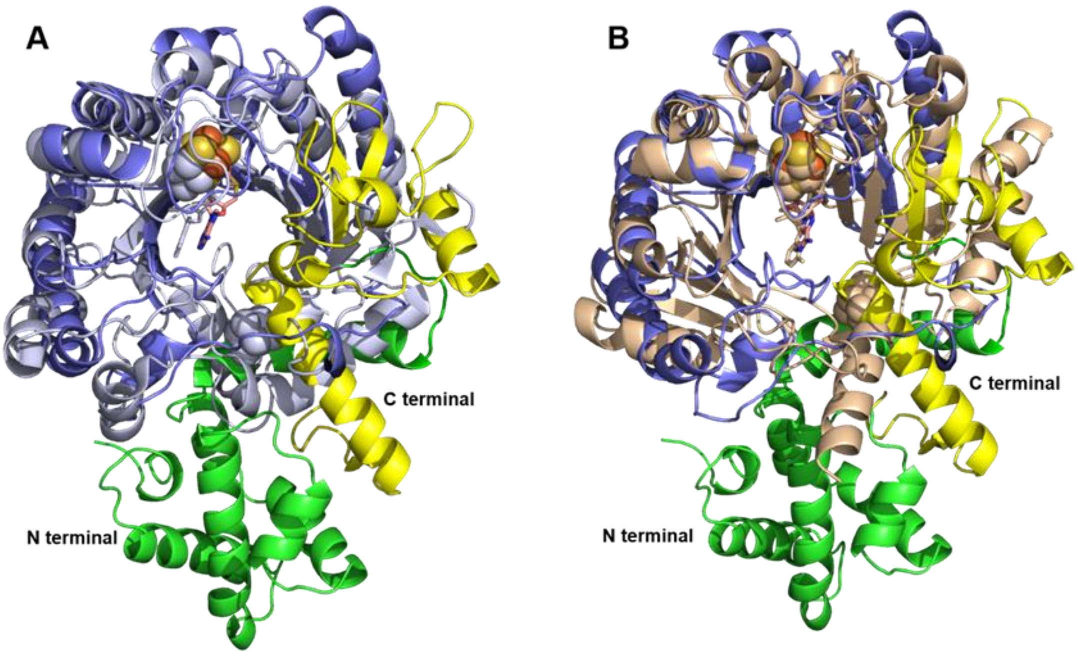

Figure S1. Structural comparison of DesII with the radical SAM structural homologs BlsE (PDB 7VOB) and MoaA (PDB 1TV8) identified by DALI search. (A) DesII (colored) compared with BlsE (light blue). (B) DesII (colored) compared with MoaA (wheat).

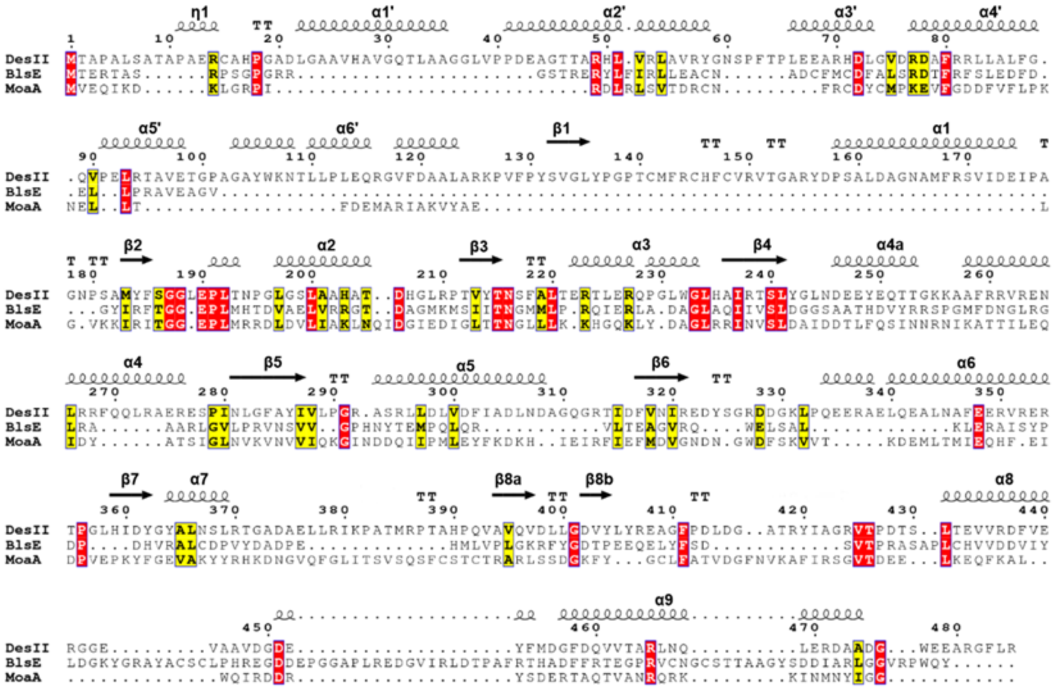

Figure S2. Sequence alignments (T-coffee web-server, default parameters): BlsE (identity 16%), MoaA (identity 15%). Image was generated with ESPript 3.0. Residues with strict identity are shown in white with a red background. Similar (> 70%) residues are shown in black with a yellow background.

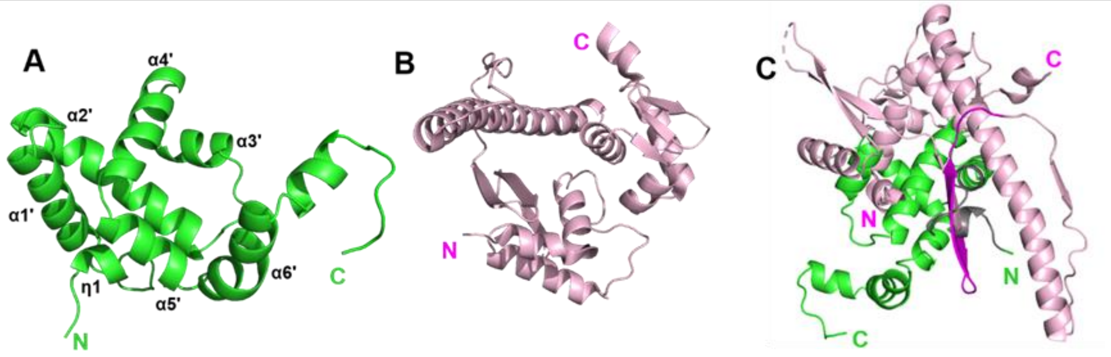

Figure S3. Structural comparison of the *N*-terminal domain of DesII and transcription factor PF0095. (A) The *N*-terminal domain of DesII (green). (B) The structure of transcription factor PF0095 (PDB 2QLZ) from *Pyrococcus furiosus* (pink). (C) Structural similarity of the *N*-terminal domain of DesII (green) and transcription factor PF0095 (magenta).

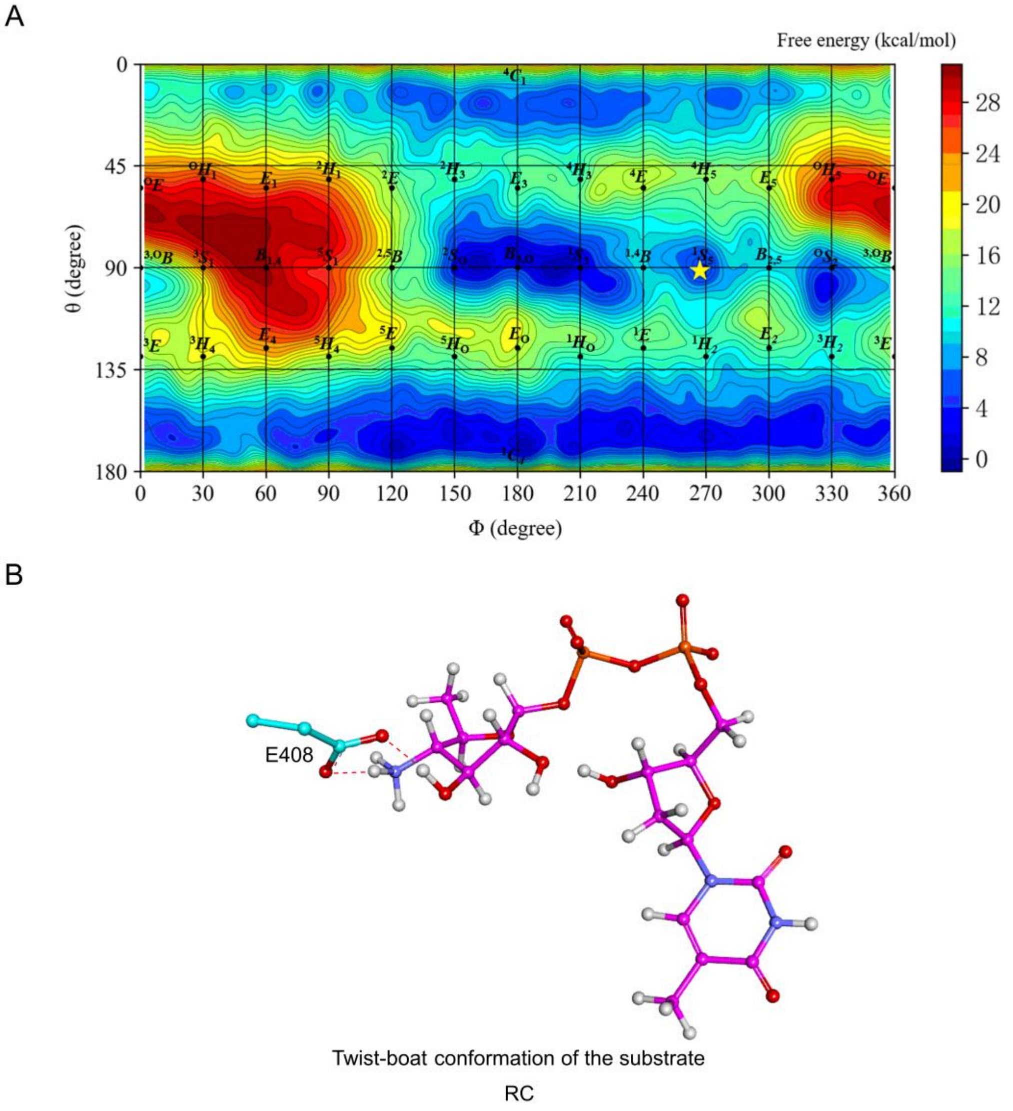

Figure S4. (A) Conformational free energy landscape with respect to Cremer-Pople ring puckering coordinates [34] for the pyranose ring of the substrate bound to DesII. (B) The twist boat conformation of the substrate (**1**) at the starred location in A from QM/MM Metadynamics simulations is the most stable conformation. The polar positions (θ ≈ 0° or 180°) correspond to a chair conformation. θ ≈ 90° and Φ ≈ 30*n* (even, integral *n*) represent boat conformations. θ ≈ 90° and Φ ≈ 30*n* (odd, integral *n*) represent twist boat conformations.

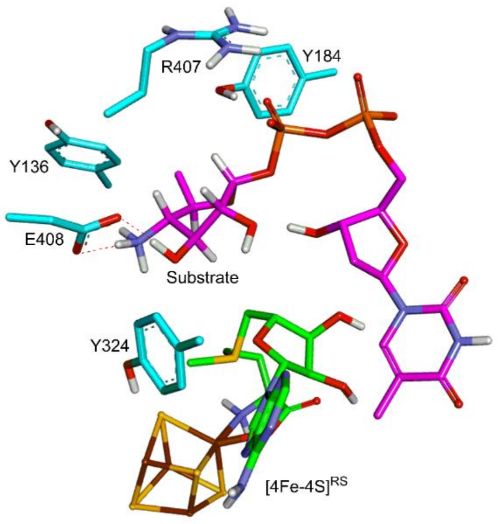

Figure S5. The QM/MM MD simulation structure of the DesII·SAM·substrate (**1**) complex. The substrate is in the twist-boat conformation.

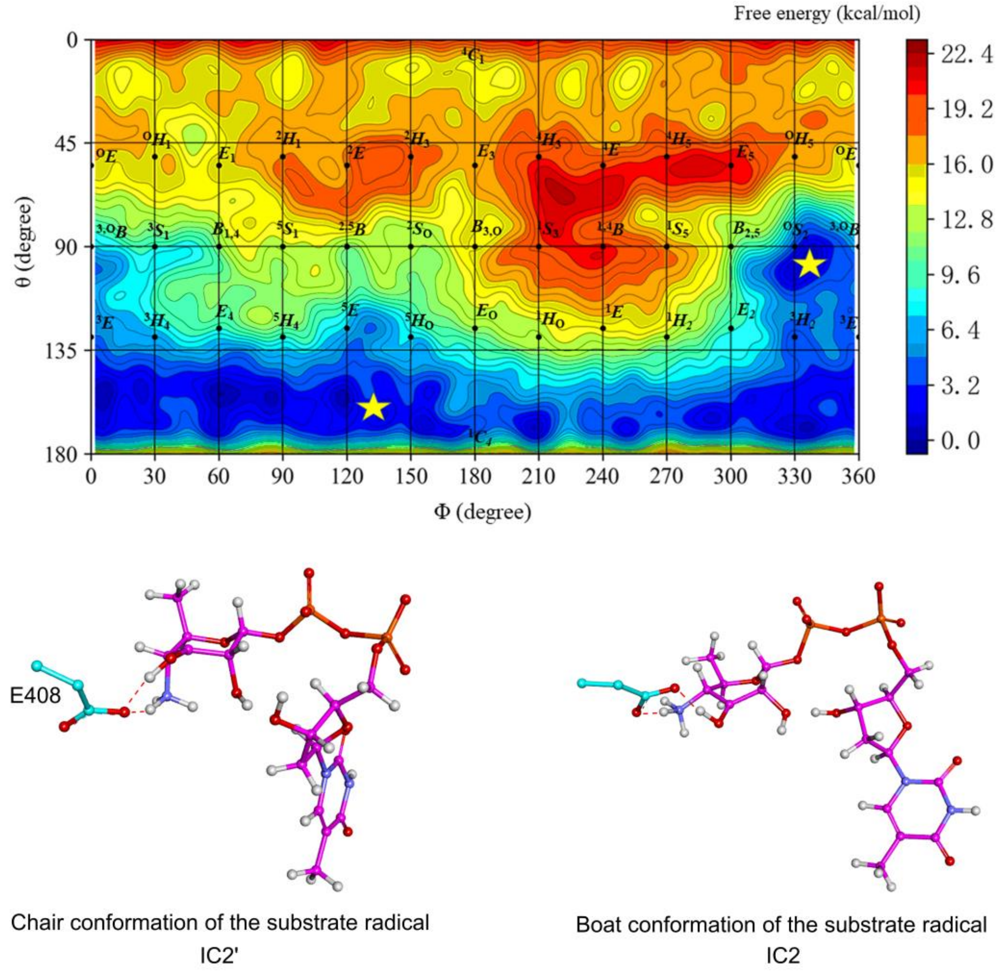

Figure S6. Conformational free energy landscape for the pyranose ring of the substrate radical intermediate **IC2** shown alongside the schematic drawings of species involved. Pyranose ring conformations are described by Cremer-Pople puckering coordinates [34] (see caption to Figure S4).

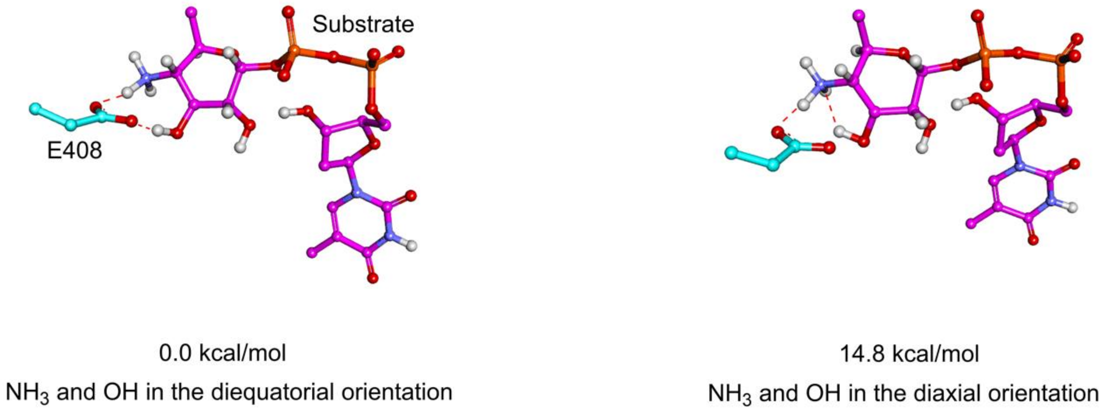

Figure S7. QM/MM calculated energies (kcal/mol) of the diequatorial orientation (double H-bonding with E408) and diaxial orientation (single H-bonding with E408).

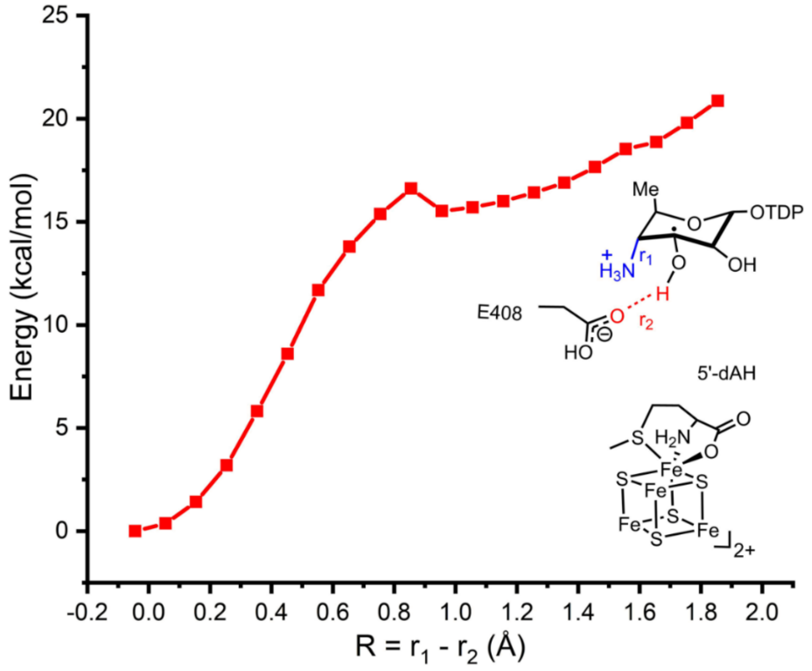

Figure S8. QM/MM scanned energy profile (kcal/mol) for deamination from the chair substrate radical. Geometry optimization of the shallow minimum along the energy profile evolves back to the initial substrate radical (**IC2′**).

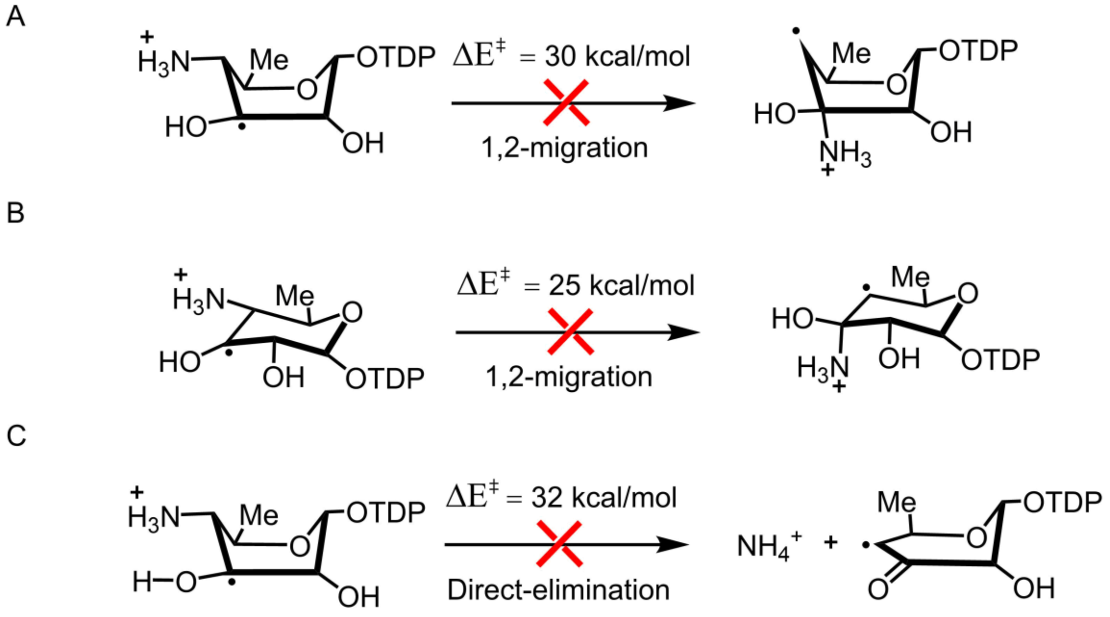

Figure S9. QM/MM calculated relative energies (kcal/mol) for different reaction pathways beginning with the substrate radical. (A) 1,2-Migration from the substrate radical in the boat conformation. (B) 1,2-Migration from the substrate radical in the chair conformation. (C) Direct elimination from the substrate radical in the boat conformation with intramolecular proton transfer.

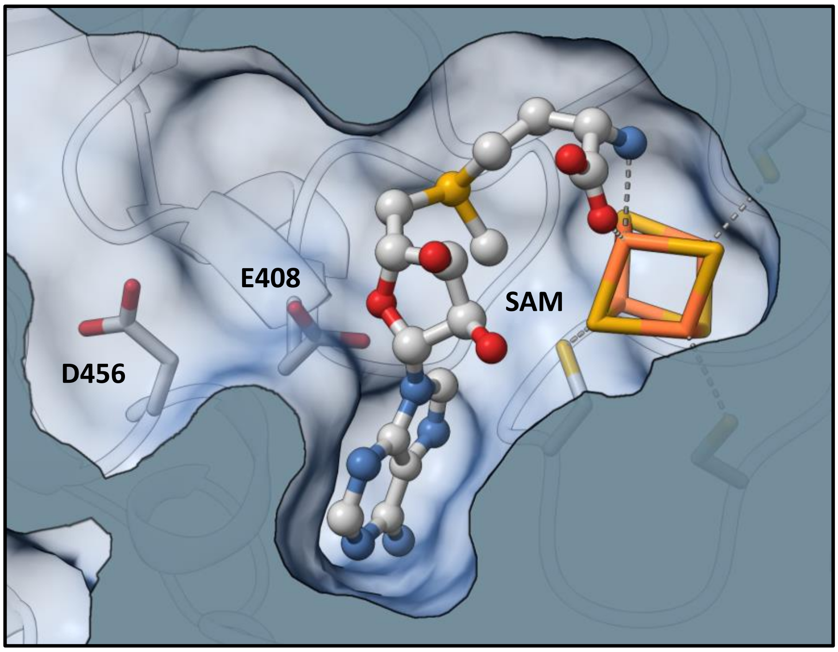

Figure S10. Cross-section analysis on the DesII active site showing the relative positioning of residues D456 and E408 with respect to SAM and the [Fe_4S_4] cluster. The active site opens towards the surrounding bulk media on the upper left coming out of the image. The image was prepared using UCSF ChimeraX. [46]

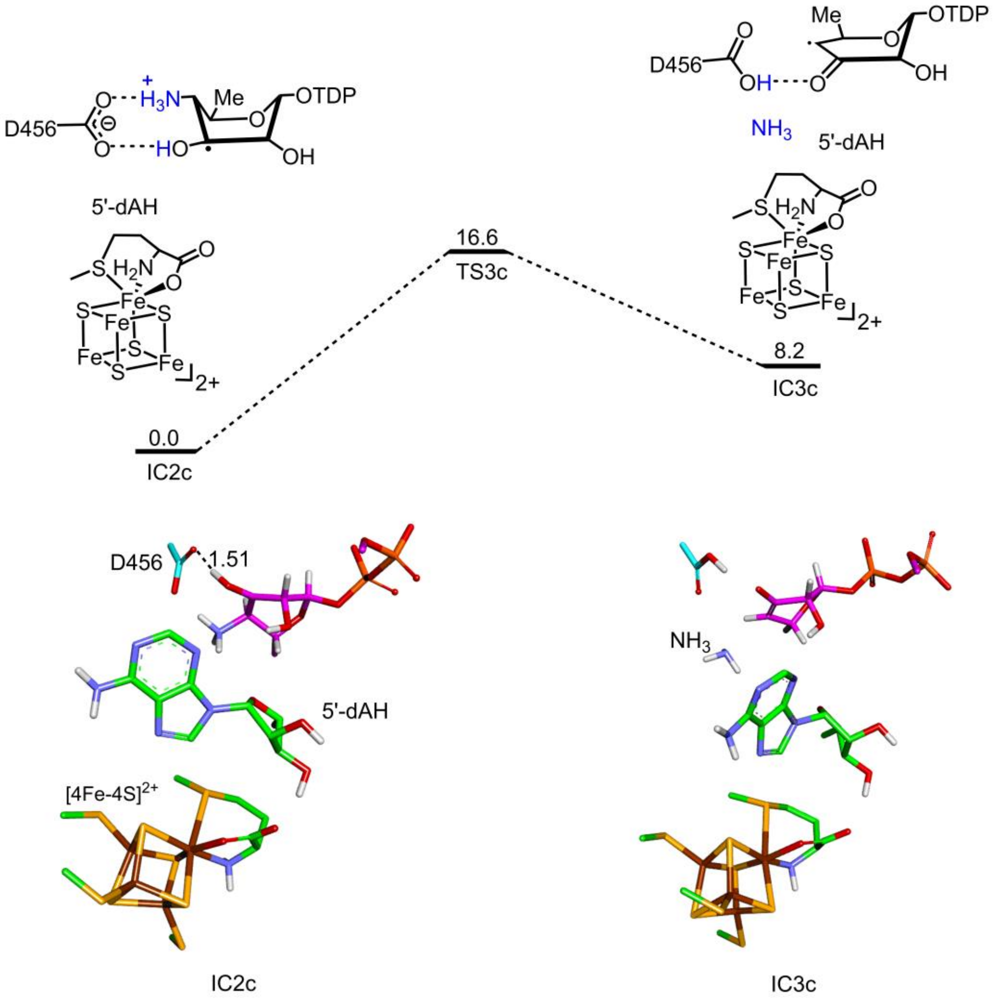

Figure S11. QM(TPSSh/def2-TZVP)/MM calculated energy profile (kcal/mol) for the deamination reaction beginning with the substrate radical in the DesII-E408A mutant active site. The TDP group of the substrate is not shown. Key distances are given in angstroms.

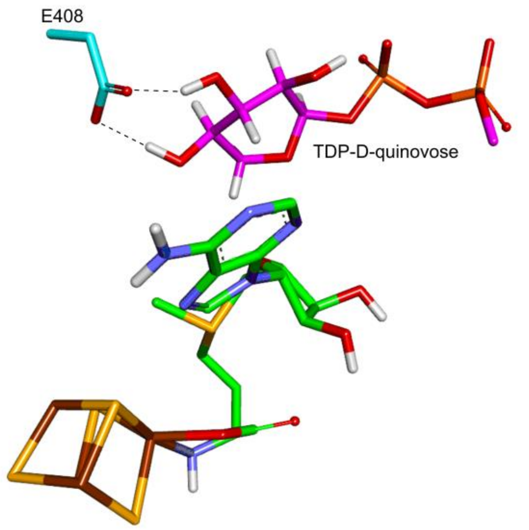

Figure S12. DesII·SAM·TDP-D-quinovose (**4**) complex. The C3′ and C4′ hydroxyl groups of the substrate form H-bonds with the carboxyl side chain of E408. The TDP group of the substrate is not shown.

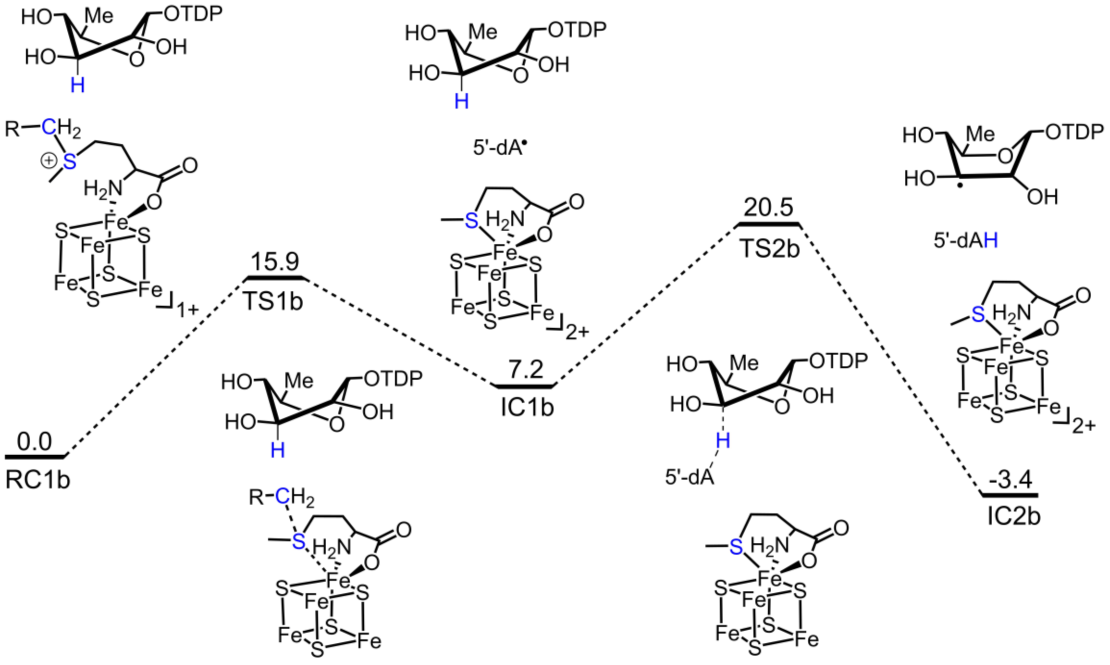

Figure S13. QM(TPSSh/def2-TZVP)/MM calculated energy profile (kcal/mol) for the reductive S−C cleavage of SAM and hydrogen atom transfer from the substrate TDP-D-quinovose (**4**).

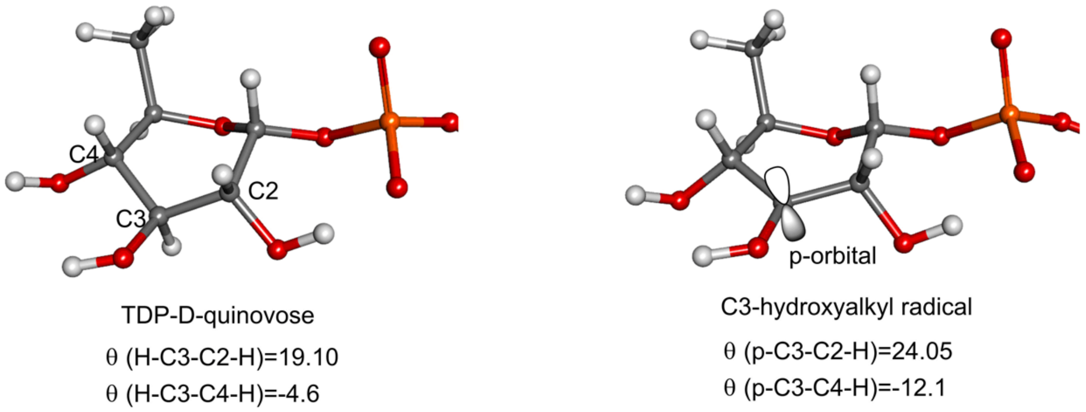

Figure S14. Calculated values of the dihedral angle θ across C2–C3 and C3–C4 for TDP-D-quinovose and the corresponding C3-hydroxyalkyl radical.

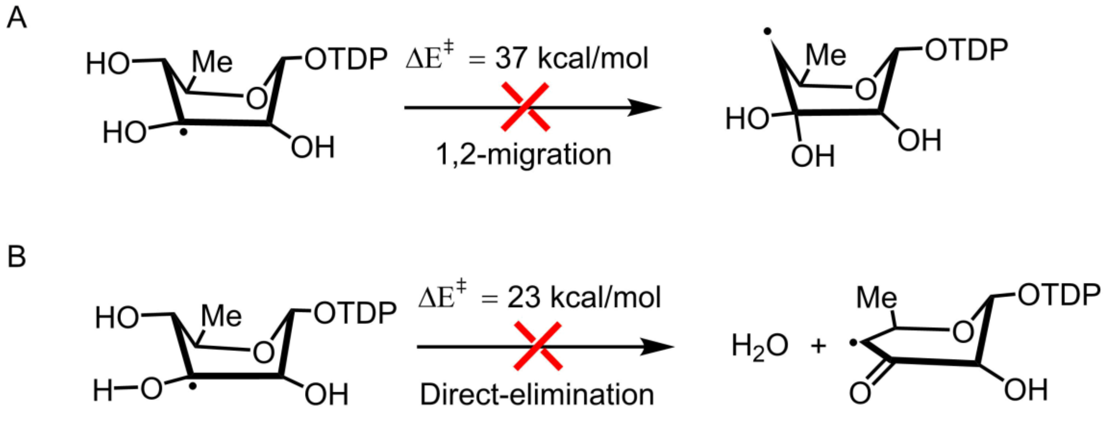

Figure S15. QM/MM calculated relative energies (kcal/mol) for different reaction pathways beginning with the substrate radical **10**. (A) 1,2-Migration from **10**. (B) Direct elimination from **10**.

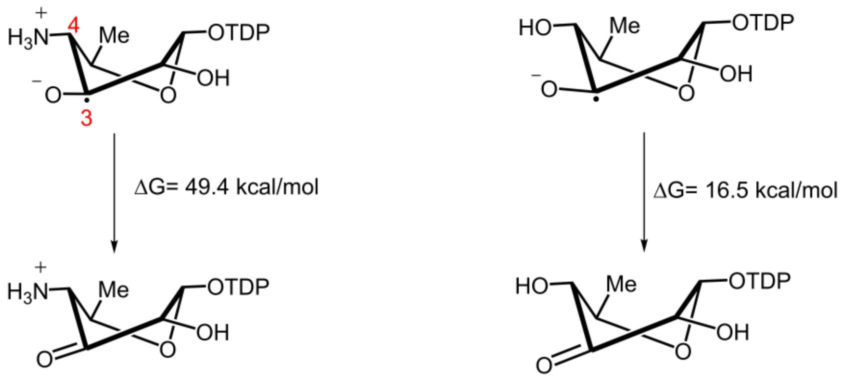

Figure S16. QM calculated relative energies (kcal/mol) for removal of an electron when C4′ carried a protonated amine versus a hydroxyl group, removal of an electron from the latter radical has a much lower energy penalty than the former.

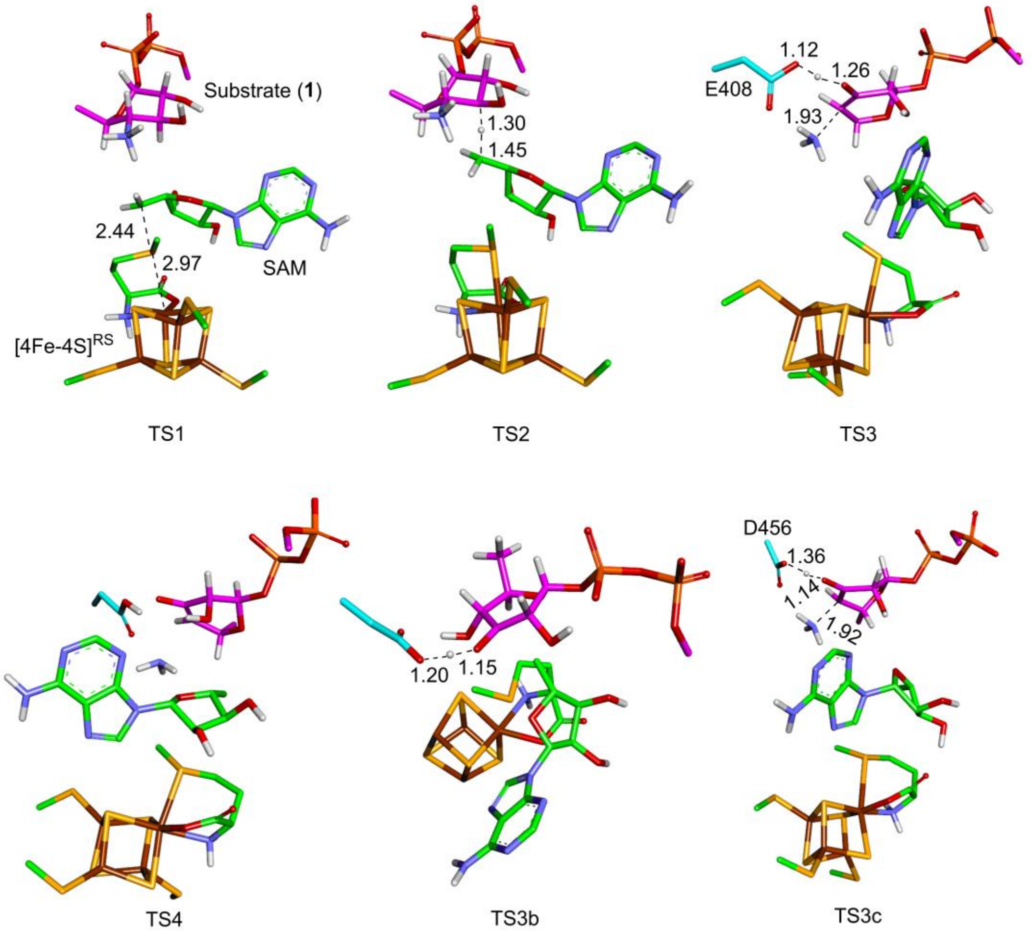

Figure S17. QM/MM calculated the transition state (TS) structures of species involved in reactions of DesII. The TDP group of the substrate is not shown. Key distances are given in angstroms.

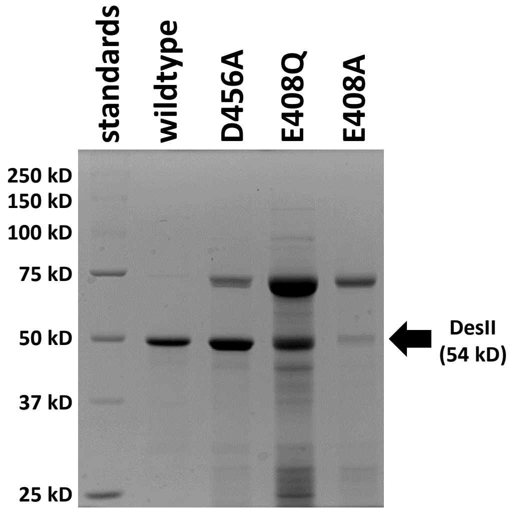

Figure S18. SDS-PAGE gel of the four DesII constructs following reconstitution of the [Fe_4S_4] cluster. Expression of the DesII-D456A mutant is poor while expression of the DesII-E408A and DesII-E408Q mutants is extremely poor. This results in significant contamination with an ca. 75 kD protein originating in the heterologous expression host (*E. coli*) that could not be easily removed with FPLC purification. Assays of the DesII-E408 mutants assumed only 20% of the total protein is DesII, though the actual concentration is likely less.

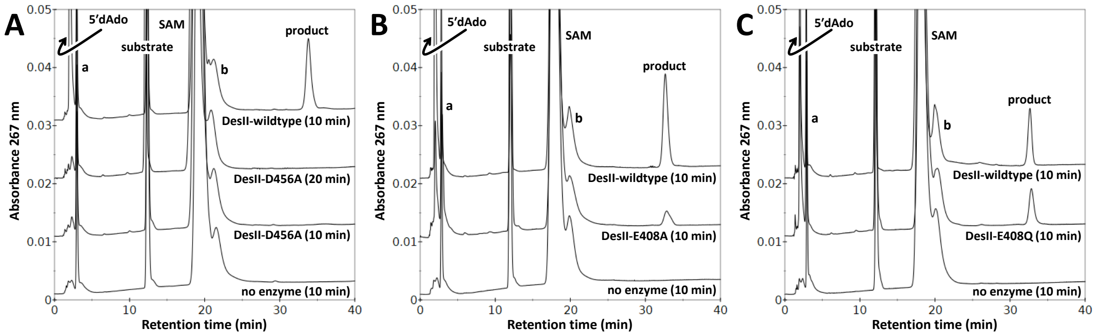

Figure S19. HPLC chromatograms demonstrating that the DesII-D456A mutant (A) is catalytically inactive whereas the DesII-E408A (B) and DesII-E408Q (C) mutants retain deaminase activity. Assays were run anaerobically in the presence of 25 mM EPPS buffer (pH 8.0, NaOH), 1 mM DTT, 500 µM SAM, 150 µM TDP-4-amino-4,6-dideoxy-D-glucose (**1**) and 4 mM $Na_2S_2O_4$ (added last). A positive control containing 1 µM DesII-wildtype was run in tandem with the mutant reactions. The concentration of the DesII-D456A enzyme was 1 µM. The concentration of the E408A and E408Q mutants is 1 µM assuming 20% of the total protein is DesII (see Figure S17). Contaminating peaks (**a** & **b**) associated with the SAM reagent are also observed.

References

[1] L. Chong, *Science* **2001**, *292*, 446-446.
[2] Y. H. Lee, X. L. Hou, R. D. Chen, J. Q. Feng, X. Liu, M. W. Ruszczycky, J. M. Gao, B. J. Wang, J. H. Zhou, H. W. Liu, *J. Am. Chem. Soc.* **2022**, *144*, 4478-4486.
[3] J. W. Zhang, X. L. Hou, Z. Chen, Y. Ko, M. W. Ruszczycky, Y. T. Chen, J. H. Zhou, H. W. Liu, *J. Am. Chem. Soc.* **2022**, *144*, 9910-9919.
[4] J. W. Pflugrath, *Acta Crystallogr. D. Biol. Crystallogr* **1999**, *55*, 1718-1725.
[5] G. Winter, D. G. Waterman, J. M. Parkhurst, A. S. Brewster, R. J. Gildea, M. Gerstel, L. Fuentes-Montero, M. Vollmar, T. Michels-Clark, I. D. Young, N. K. Sauter, G. Evans, *Acta Crystallogr. D Struct. Biol.* **2018**, *74*, 85-97.
[6] C. Vonrhein, C. Flensburg, P. Keller, A. Sharff, O. Smart, W. Paciorek, T. Womack, G. Bricogne, *Acta Crystallogr. D Struct. Biol.* **2011**, *67*, 293-302.
[7] P. D. Adams, P. V. Afonine, G. Bunkóczi, V. B. Chen, I. W. Davis, N. Echols, J. J. Headd, L. W. Hung, G. J. Kapral, R. W. Grosse-Kunstleve, A. J. McCoy, N. W. Moriarty, R. Oeffner, R. J. Read, D. C. Richardson, J. S. Richardson, T. C. Terwilliger, P. H. Zwart, *Acta Crystallogr. D Struct. Biol.* **2010**, *66*, 213-221.
[8] P. Emsley, B. Lohkamp, W. G. Scott, K. Cowtan, *Acta Crystallogr. D. Biol. Crystallogr* **2010**, *66*, 486-501.
[9] R. A. Laskowski, M. W. Macarthur, D. S. Moss, J. M. Thornton, *J. Appl. Cryst.* **1993**, *26*, 283-291.
[10] I. W. Davis, A. Leaver-Fay, V. B. Chen, J. N. Block, G. J. Kapral, X. Wang, L. W. Murray, W. B. Arendall, 3rd, J. Snoeyink, J. S. Richardson, D. C. Richardson, *Nucleic Acids Res.* **2007**, *35*, W375-383.
[11] D. Seeliger, B. L. de Groot, *J. Comput. Aided Mol. Des.* **2010**, *24*, 417-422.
[12] L. Holm, C. Sander, *Trends Biochem. Sci.* **1995**, *20*, 478-480.
[13] P. Hänzelmann, H. Schindelin, *Proc. Natl. Acad. Sci. U.S.A.* **2004**, *101*, 12870-12875.
[14] P. Hanzelmann, H. Schindelin, *Proc. Natl. Acad. Sci. U.S.A.* **2006**, *103*, 6829-6834.
[15] K. Yokoyama, D. Li, H. Pang, *ACS Bio. Med. Chem. Au* **2022**, *2*, 94-108.
[16] P. J. Goldman, T. L. Grove, S. J. Booker, C. L. Drennan, *Proc. Natl. Acad. Sci. U.S.A.* **2013**, *110*, 15949-15954.
[17] T. A. J. Grell, P. J. Goldman, C. L. Drennan, *J. Biol. Chem.* **2015**, *290*, 3964-3971.
[18] C. R. Sondergaard, M. H. M. Olsson, M. Rostkowski, J. H. Jensen, *J. Chem. Theory Comput.* **2011**, *7*, 2284-2295.
[19] P. F. Li, K. M. Merz, *J. Chem. Inf. Model.* **2016**, *56*, 599-604.
[20] P. F. Li, K. M. Merz, *Chem. Rev.* **2017**, *117*, 1564-1686.
[21] J. A. Maier, C. Martinez, K. Kasavajhala, L. Wickstrom, K. E. Hauser, C. Simmerling, *J. Chem. Theory Comput.* **2015**, *11*, 3696-3713.
[22] J. M. Wang, R. M. Wolf, J. W. Caldwell, P. A. Kollman, D. A. Case, *J. Comput. Chem.* **2004**, *25*, 1157-1174.
[23] C. I. Bayly, P. Cieplak, W. D. Cornell, P. A. Kollman, *J. Phys. Chem.* **1993**, *97*, 10269-10280.
[24] J. VandeVondele, M. Krack, F. Mohamed, M. Parrinello, T. Chassaing, J. Hutter, *Comput. Phys. Commun.* **2005**, *167*, 103-128.
[25] T. Laino, F. Mohamed, A. Laio, M. Parrinello, *J. Chem. Theory Comput*. **2005**, *1*, 1176-1184.
[26] A. Laio, J. VandeVondele, U. Rothlisberger, *J. Chem. Phys.* **2002**, *116*, 6941-6947.
[27] J. VandeVondele, J. Hutter, *J. Chem. Phys.* **2007**, *127*.
[28] S. Goedecker, M. Teter, J. Hutter, *Phys. Rev. B* **1996**, *54*, 1703-1710.
[29] M. Guidon, J. Hutter, J. VandeVondele, *J. Chem. Theory Comput.* **2010**, *6*, 2348-2364.
[30] A. W. Götz, M. A. Clark, R. C. Walker, *J. Comput. Chem.* **2014**, *35*, 95-108.
[31] F. Neese, *WIREs Comput. Mol. Sci.* **2012**, *2*, 73-78.
[32] G. A. Tribello, M. Bonomi, D. Branduardi, C. Camilloni, G. Bussi, *Comput. Phys. Commun.* **2014**, *185*, 604-613.
[33] C. Bannwarth, S. Ehlert, S. Grimme, *J. Chem. Theory Comput.* **2019**, *15*, 1652-1671.
[34] D. Cremer, J. A. Pople, *J. Am. Chem. Soc.* **1975**, *97*, 1354-1358.
[35] A. H. de Vries, P. Sherwood, S. J. Collins, A. M. Rigby, M. Rigutto, G. J. Kramer, *J. Phys. Chem.* **1999**, *103*, 6133-6141.
[36] S. Metz, J. Kästner, A. A. Sokol, T. W. Keal, P. Sherwood, *WIREs Comput. Mol. Sci.* **2014**, *4*, 101-110.
[37] R. Ahlrichs, M. Bar, M. Haser, H. Horn, C. Kolmel, *Chem. Phys. Lett.* **1989**, *162*, 165-169.
[38] W. Smith, C. W. Yong, P. M. Rodger, *Mol. Simul.* **2002**, *28*, 385-471.
[39] D. Bakowies, W. Thiel, *J. Phys. Chem.* **1996**, *100*, 10580-10594.
[40] S. Grimme, *J. Comput. Chem.* **2006**, *27*, 1787-1799.
[41] S. Grimme, J. Antony, S. Ehrlich, H. Krieg, *J. Chem. Phys.* **2010**, *132*.
[42] S. Grimme, S. Ehrlich, L. Goerigk, *J. Comput. Chem.* **2011**, *32*, 1456-1465.
[43] P. H. Szu, X. M. He, L. S. Zhao, H. W. Liu, *Angew. Chem. Int. Ed.* **2005**, *44*, 6742-6746.
[44] P. H. Szu, M. W. Ruszczycky, S. H. Choi, F. Yan, H. W. Liu, *J. Am. Chem. Soc.* **2009**, *131*, 14030-14042.
[45] L. S. Zhao, S. Borisova, S. M. Yeung, H. W. Liu, *J. Am. Chem. Soc.* **2001**, *123*, 7909-7910.
[46] E. F. Pettersen, T. D. Goddard, C. R. C. Huang, E. E. C. Meng, G. S. Couch, T. I. Croll, J. H. Morris, T. E. Ferrin, *Protein Sci.* **2021**, *30*, 70-82.
